# Comparison of the mutational profiles of neuroendocrine breast tumours, invasive ductal carcinomas and pancreatic neuroendocrine carcinomas

**DOI:** 10.1038/s41389-022-00427-1

**Published:** 2022-09-09

**Authors:** Peeter Karihtala, Katja Porvari, Nelli Roininen, Sari Voutilainen, Johanna Mattson, Päivi Heikkilä, Kirsi-Maria Haapasaari, Katri Selander

**Affiliations:** 1 Department of Oncology, Helsinki University Hospital Comprehensive Cancer Center and University of Helsinki, Helsinki, Finland; 2 Department of Pathology, Medical Research Center Oulu, Oulu University Hospital and University of Oulu, Oulu, Finland; 3 Department of Oncology and Radiotherapy, Medical Research Center Oulu, Oulu University Hospital and University of Oulu, Oulu, Finland; 4 Department of Pathology, University of Helsinki and Helsinki University Hospital, Helsinki, Finland

**Keywords:** Breast cancer, Cancer genetics, Cancer genetics, Oncogenes

## Abstract

The pathophysiology and the optimal treatment of breast neuroendocrine tumours (NETs) are unknown. We compared the mutational profiles of breast NETs (*n* = 53) with those of 724 publicly available invasive ductal carcinoma (IDC) and 98 pancreatic NET (PNET) cases. The only significantly different pathogenetic or unknown variant rate between breast NETs and IDCs was detected in the *TP53* (11.3% in breast NETs and 41% in IDCs, adjusted *p* value 0.027) and *ADCK2* (9.4% in breast NETs vs. 0.28% in IDCs, adjusted *p* value 0.045) genes. Between breast NETs and PNETs, different pathogenetic or unknown variant frequencies were detected in 30 genes. For example, *MEN1* was mutated in only 6% of breast NETs and 37% in PNETs (adjusted *p* value 0.00050), and *GATA3* pathogenetic or unknown variants were only found in 17.0% of breast NETs and 0% in PNETs (adjusted *p* value 0.0010). The most commonly affected oncogenic pathways in the breast NET cases were *PI3K/Akt/mTOR*, *NOTCH* and *RTK-RAS* pathways. Breast NETs had typically clock-like mutational signatures and signatures associated with defective DNA mismatch repair in their mutational landscape. Our results suggest that the breast NET mutational profile more closely resembles that of IDCs than that of PNETs. These results also revealed several potentially druggable targets, such as MMRd, in breast NETs. In conclusion, breast NETs are indeed a separate breast cancer entity, but their optimal treatment remains to be elucidated.

## Introduction

Recently, the International Agency for Research on Cancer and the World Health Organization (WHO) agreed to adopt the term ‘neuroendocrine neoplasm’ (NEN) to encompass all tumour classes with predominant neuroendocrine differentiation [1, 2]. Although NENs most commonly originate from abdominal organs, they may arise from virtually any anatomical site, including the breast. In the latest WHO classification, NENs were further divided into neuroendocrine tumours (NETs) and neuroendocrine carcinomas (NECs) [1]. The reported incidence of breast NETs in most studies ranges between 0.1 and 5%, mostly depending on the diagnostic criteria used and screening of breast tumours with diagnostic neuroendocrine immunostainings, synaptophysin and chromogranin A [1, 3–5]. Breast-originating NECs demonstrate morphological characteristics of small-cell and large-cell carcinomas of the lung and are extremely rare [1].

Breast NETs probably result from an early divergent differentiation of breast cancer stem cells into both neuroendocrine and epithelial lines [1]. The essential criteria for diagnosing breast NETs are specific histological features and immune-profile characteristics of neuroendocrine differentiation, while coexisting ductal carcinomas in situ is a desirable criterion [1]. We and others have previously reported that breast NETs occur generally in older women, they are almost solely oestrogen receptor (ER)-positive and human epidermal growth factor 2 (HER2)-negative, and their prognosis is slightly poorer than that of invasive ductal carcinomas (IDCs) of the breast [4–7]. The main clinical problem is the absence of virtually any trial-based evidence on how breast NETs should be optimally treated.

Based on previous studies on breast NET pathogenetic or unknown variants (‘mutations’) [8–10], we performed whole exome sequencing from a cohort of 53 breast NETs and collected their comprehensive clinical and pathological data. We compared the sequencing results with previously published mutational spectra of IDC and pancreatic NET (PNET) cases from The Cancer Genomic Atlas (TCGA) [11, 12]. Our results suggest that the mutational profile of breast NETs is rather similar to that of IDCs.

## Materials and methods

### Clinical and pathological data

We collected data retrospectively on 53 patients diagnosed with breast NET from the Oulu University Hospital and Helsinki University Hospital from January 2006 to December 2018. The diagnoses were re-reviewed to meet the latest WHO criteria for breast NETs [1]. Only cases showing extensive (>50% of tumour cells) synaptophysin or chromogranin A expression were included. Mucinous carcinomas were excluded, even if they demonstrated neuroendocrine marker expression. To exclude NECs, breast NENs with small-cell or large-cell features were not included in the current dataset. To exclude carcinomas of extramammary origin, the breast NET cases without [1] abdominal and thoracic imaging at the time of diagnosis or without [2] histological identification of a coexisting IDC component were excluded. There was no evidence of extramammary primary NETs during the follow-up.

The patients were grouped according to the tumour, node and metastasis classification [13]. ER, progesterone receptor (PR) and Ki-67 expressions were studied by immunohistochemistry as described previously [14]. HER2 expression was studied by immunohistochemistry, and when an HER2-positive result was recorded, gene amplification status was determined using chromogenic in situ hybridisation. Cancers with six or more gene copies were considered HER2-positive.

### DNA isolation, exome capture and sequencing

Genomic DNA was isolated using the QIAamp DNA FFPE Tissue Kit and automated QIAcube sample preparation instrument according to the manufacturer’s protocol (Qiagen, Hilden, Germany). Exome capture was accomplished using the Agilent SureSelect Human Clinical Research Exome v2 QXT capture kit (Agilent, CA, USA). Briefly, the genomic DNA was subjected to tagmentation reactions, inserting adaptor sequences randomly throughout the genome. The DNA was PCR-amplified and then incubated with biotin-labelled RNA capture probes complementary to every exon. Following the purification of the exonic sequences through streptavidin-magnetic bead separation, the DNA was amplified with primers that introduced an 8-nucleotide index so that separate samples could be run in the same lane for sequence analysis. The exomic libraries were run on the NextSeq500 next-generation sequencer from Illumina (Illumina, San Diego, CA) with paired-end 75 base pair reads.

### Invasive ductal carcinoma (IDC) data

Open-access Masked Somatic Mutation data of the TCGA breast cancer cohort (TCGA-BRCA) were downloaded from the Genomic Data Commons (GDC) [11] using the R package *TCGAbiolinks* [15], v. 2.16.4. Harmonised data (aligned to the hg38 reference genome), which were generated using a Mutect2 variant calling pipeline, were downloaded and used for the analyses. The *TCGAquerysubtype* function was used for retrieving additional annotations of molecular subtypes, namely gene expression-based PAM50 subtypes [16]. The R package *Maftools* [17], v. 2.4.12, was used for reading data into R and combining the clinical data with the variant data. Data were filtered to include only samples with the primary diagnosis type ‘Infiltrating duct carcinoma, NOS’.

### Pancreatic neuroendocrine tumour (PNET) data

Publicly available data concerning the coding substitutions and indels present in 98 PNETs were used in this study. These somatic pathogenetic or unknown variant data were accessed from a publication on PNET mutational landscapes [12].

### Data quality check and alignment

The breast NET pathogenetic or unknown variant analysis began with raw sequencing data in the FASTQ format. Quality and adapter trimming were carried out via TrimGalore, v. 0.6.4, in paired-end mode using Cutadapt, v. 2.4 [17]. A PHRED score cut-off of 20 was used, with any reads falling outside of this threshold being discarded. Only sequence reads over 20 base pairs in length that had an error rate of less than 10% were retained for further analysis. The quality of raw and trimmed reads was inspected using FastQC, v. 0.11.9 [18]. The trimmed reads were aligned to the human reference genome GRCh38 using the BWA aligner, v. 0.7.17 [19].

### Somatic variant calling

Pathogenetic or unknown variants were called using the Mutect2 variant caller in the GATK workflow, v. 4.0 [20]. A panel of normals, consisting of exomes from the 1000 genome dataset [21], was used.

### Variant annotation and filtering

In order to filter out likely artefacts and sequencing errors from the initial set of candidate pathogenetic or unknown variants, the Mutect2-evaluated metrics were used (Supplementary Table 1). The Mutect2-filtered variants were annotated using Ensembl Variant Effect Predictor (VEP), v. 100.2 [22]. Based on the population databases included in the VEP (Supplementary Table 2), a preliminary population allele frequency filter was applied by requiring an allele frequency of 0.01 or below in all listed population databases and subsets thereof. Moreover, variants were filtered based on their predicted impact assigned by VEP such that only moderate- or high-impact variants were retained.

To overcome the lack of normal control tissue, further filtering steps involving population allele frequencies and variant effect predictions were applied to this preliminary set of somatic variant calls (Supplementary Table 3). The final filtered variant set of 4330 variants in 3112 genes was obtained by filtering the preliminary call set based on the prediction scores of the Cancer-specific High-throughput Annotation of Somatic Mutations (CHASM-3.1) [23], and the Variant Effect Scoring Tool (VEST-4) [24, 25], and by further manually excluding two variants that were deemed as very likely germline variants. These two variants were single-nucleotide polymorphisms (SNPs): D159N in the gene CBWD6 and R296T in the gene LILRA1. Both of these variants are annotated in the dbSNP database (IDs rs62555254 and rs757520653), were flagged by MuTect2 as ‘germline_risk’ and were present in multiple patients (6 and 14, respectively). The predictions of *CHASM-3.1* and *VEST-4* were obtained using programmatic access to *CRAVAT*: Cancer-Related Analysis of Variants Toolkit [25]. The *CHASM* method is based on a random forest classifier and is used for identifying and prioritising those missense pathogenetic or unknown variants that are most likely to generate functional changes that enhance tumour cell proliferation. The *VEST-4* method is also a random forest-based method for prioritising rare missense and indel variants with likely involvement in human disease. Both methods generated pathogenicity scores ranging from 0 to 1, with 1 indicating likely pathogenic/driver pathogenetic or unknown variant and 0 indicating likely non-pathogenic/passenger pathogenetic or unknown variant.

### Cohort comparisons

The *Maftools* function *mafCompare* was used for analysing differentially mutated genes between two cancer cohorts. Genes with a minimum of five mutated samples in at least one cohort were considered in the analysis. Differentially mutated genes were detected by performing Fisher’s exact test on each gene between the two groups.

### Variant associations with clinicopathological data

The association of the breast NET cohort variants with binary clinicopathological variables was studied by subsetting the cohort into subgroups using the *subsetMaf* function of *Maftools* based on binary clinicopathological variable statuses. The variables under study included tumour size, nodal status, the presence of distant metastases at diagnosis, the multifocality of cancer, ER or PR expression, Ki-67 expression, HER2 amplification, immunohistochemical synaptophysin or chromogranin expression, number of deliveries, and primary tumour size in millimetres. Differentially mutated genes between the subgroups were assessed using *mafCompare* as described above in the *Cohort Comparisons* section, except that a minimum of two mutated samples in at least one subgroup was required.

Variant associations with non-binary clinical variables—that is, age at diagnosis, number of births, and serum chromogranin A levels—were analysed by comparing the distributions of the variable values in groups based on the pathogenetic or unknown variant statuses of the mutated genes. The R package *ggpubr*, v. 0.4.0 [26], was used for visualising the data distributions of the subgroups as boxplots for the 30 most mutated genes. A statistical comparison of the variable distributions regarding pathogenetic or unknown variant statuses of all mutated genes in the filtered breast NET variant set was performed with a *t*-test (for data with normal distribution) or a Mann–Whitney *U*-test (if the data were not normally distributed). The Shapiro–Wilk normality test was used for assessing the distributions of the five variable values. The *p* values were corrected for multiple tests using the Benjamini–Hochberg procedure [27].

### Survival analysis

Survival analysis of patients grouped by pathogenetic or unknown variant statuses was performed using R packages *survival*, v. 3.2-7 [28], and *survminer*, v. 0.4.8 [26]. Death due to breast cancer was used as the endpoint event, with all other patients considered as censored cases. Of all the mutated genes in the final filtered variant set, those mutated in at least two patients were used in the assessment. The *survdiff* function was used for statistically testing the survival curve differences of the non-mutated and mutated patient groups for each gene, and the *ggsurvplot* function was subsequently used for plotting the Kaplan–Meier survival curves of the comparisons that had a *p* value < 0.05.

### Somatic interactions

Mutually exclusive or co-occurring pairs of mutated genes within the breast NET cohort were detected using the *somaticInteractions* function of *Maftools*. This function performed a pairwise Fisher’s exact test to detect such interactions among the top 30 most mutated genes in the cohort.

### Oncogenic signalling pathway enrichment analysis

The *OncogenicPathways* function of *Maftools* was used for checking for the enrichment of known oncogenic pathways [29] in the breast NET, IDC and PNET cohorts. This function calculated fractions of affected genes in a pathway and fractions of samples with pathogenetic or unknown variants in pathway genes.

### Pathogenetic or unknown variant statistics of genes across all TCGA cancer types

Open-access Masked Somatic Mutation data of all TCGA cohorts were downloaded from the GDC as described above. *Maftools* [30], v. 2.4.12, and custom R scripts were used for reading somatic variant data of all TCGA cohorts into R and combining the clinical data with the variant data. For each cancer type, the number and percentage of samples having pathogenetic or unknown variants in the genes that were found to be mutated in one or more TCGA data sets were calculated using custom R scripts.

### Mutational signature analysis

Mutational signatures were extracted from the filtered somatic variants of the breast NET cohort using two separate tools: the R packages *Maftools* [30], v. 2.4.12, and SigProfiler [31], v. 3.1. *Maftools* only extracts single-base-substitution (SBS) signatures and estimates the similarity of each signature to known COSMIC signatures separately. SigProfiler allows the extraction of SBS and indel (ID) signatures, decomposing the de novo extracted signatures as a weighted sum of COSMIC signatures, and further fitting those COSMIC signatures back to individual patients’ mutational spectra. SigProfiler was thus used to extract, decompose and refit mutational signatures in the breast NET cohort and, additionally, the PNET and IDC cohorts. Further details on the methods of mutational signature analyses are given in the Supplementary data.

ADCK2 mRNA and immunostaining analyses have been reported in the Supplementary data.

### Ethical approval

This study was approved by the Local Ethics Committee of the Ostrobothnian Hospital District (114/2011, amendment 23.2.2015) and the National Supervisory Authority for Welfare and Health (1339/05.01.00.06/2009).

## Results

Our final cohort included 53 patients fulfilling the most recent WHO criteria for breast NETs [1]. At the time of diagnosis, four patients had distant metastases, which were all bone metastases (Tables 1 and 2). The mean follow-up time was 41.9 months (95% CI 33.6–50.3 months). In 24 patients (45.3% of evaluable cases), breast NETs were either suspected or diagnosed based on a diagnostic needle biopsy.

**Table 1:** Clinical and pathological baseline characteristics of the breast neuroendocrine tumour (NET) cohort.

T class	
T1	32 (60.4%)
T2	16 (30.2%)
T3	3 (5.7%)
T4	2 (3.8%)
Mean size of primary tumour, mm (95% confidence interval)	26.8 (21.3–32.3)
N class	
N0	30 (58.8%)
N1	11 (20.8%)
N2	8 (15.1%)
N3	2 (3.8%)
Missing	2 (3.8%)
Primary distant metastases	
Yes	4 (7.5%)
No	49 (92.5%)
Multifocal cancer	
Yes	13 (24.5%)
No	40 (75.5%)
HER2 amplification	
Negative	51 (96.2%)
Positive	2 (3.8%)
Oestrogen receptor expression	
Negative (0%)	2 (3.8%)
Low (1–9%)	0 (0%)
Moderate (10–59%)	0 (0%)
High (>60%)	51 (96.2%)
Progesterone receptor expression	
Negative (0%)	2 (3.8%)
Low (1–9%)	5 (9.4%)
Moderate (10–59%)	8 (15.1%)
High (>60%)	34 (64.2%)
Ki-67 expression	
Negative (<5%)	1 (1.9%)
Low (5–14%)	16 (30.2%)
Moderate (15–30%)	19 (35.8%)
High (>30%)	17 (32.1%)
In situ carcinoma present	
Yes	36 (67.9%)
No	17 (32.1%)
Synaptophysin expression	
Yes	53 (100%)
No	0 (0%)
Chromogranin expression	
Yes	33 (62.3%)
No	14 (26.4%)
Not available	6 (11.3%)
Menopausal status	
Postmenopausal	49 (92.5%)
Premenopausal	3 (5.7%)
Unknown	1 (1.9%)

**Table 2:** The primary treatment modalities of the breast neuroendocrine tumour (NET) cohort.

Surgical management	
Partial breast resection and axillary evacuation	4 (7.5%)
Mastectomy and axillary evacuation	21 (39.6%)
Partial breast resection and sentinel node biopsy	18 (34.0%)
Mastectomy and sentinel node biopsy	8 (15.1%)
Partial breast resection only	1 (1.9%)
Mastectomy only	1 (1.9%)
Adjuvant chemotherapy	
FEC	4 (7.5%)
Docetaxel and FEC	10 (18.9%)
Other adjuvant chemotherapy	2 (3.8%)
Neoadjuvant chemotherapy	2 (3.8%)
No adjuvant chemotherapy	35 (66.0%)
Adjuvant trastuzumab	
Yes	2 (3.8%)
No	51 (96.2%)
Adjuvant radiotherapy	
Yes	37 (69.8%)
No	16 (30.2%)
Adjuvant endocrine therapy	
Tamoxifen	7 (13.2%)
Aromatase inhibitor	36 (67.9%)
No adjuvant endocrine therapy	10 (18.9%)

*FEC* 5-fluorouracil, epirubicin and cyclophosphamide.

### Explorative analysis of variants

**Fig. 1:**
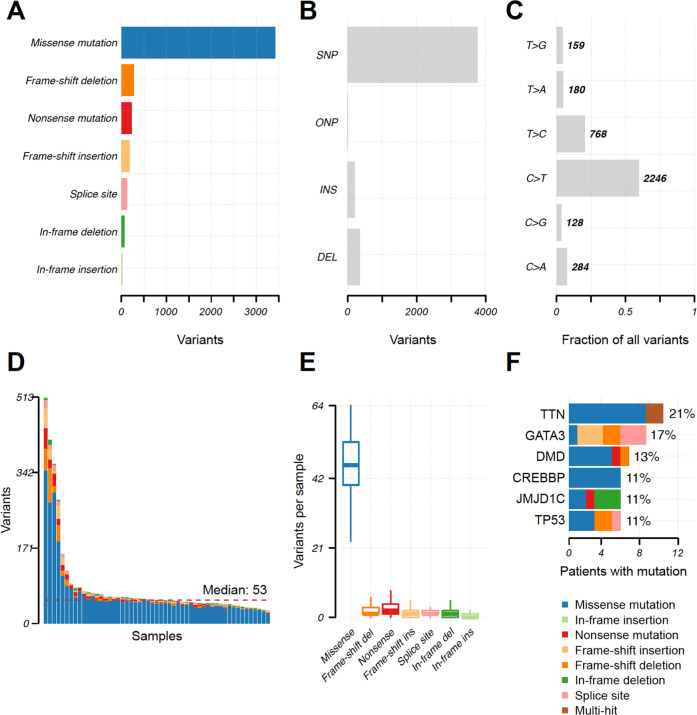
Summary of the filtered breast NET cohort variants. Panels **A**–**C** show the distribution of variants. Panel **D** shows the number of variants in each sample. In panel **E**, variants per sample are demonstrated and panel **F** shows the six most common pathogenetic or unknown variants in the breast NET cohort. TTN variants are likely not associated with cancer. SNP single-nucleotide variant, ONP oligo-nucleotide polymorphism, INS insertion, DEL deletion.

The filtered variant set consisting of 4330 variants in 3112 genes was stored in the Mutation Annotation Format, and the properties of the variants were summarised and visualised (Fig. 1A–C). Variants were most commonly missense pathogenetic or unknown variants and SNPs, with C > T pathogenetic or unknown variants as the predominant single-nucleotide variant class. The number of pathogenetic or unknown variants per patient ranged from 30 to 513, with a median of 53 (Fig. 1D). Of the 30 most mutated genes, TTN and PLEC were flagged by *Maftools* as genes frequently found to be mutated in exome studies due to their length, and they are thus likely to be passengers. They were not, however, excluded from the call set at this point. In addition, the most frequently mutated genes in this cohort were *MYCBP2*, *GATA3*, *SCN5A*, *DMD*, *EP400*, *TP53*, *JMJD1C* and *CREBBP*, but the pathogenetic or unknown variant compositions per gene varied substantially. For example, 10 of the top 30 mutated genes had only missense pathogenetic or unknown variants; while in the nine patients with GATA pathogenetic or unknown variants, four different variant types were observed. (Fig. 1E, F and Supplementary Fig. 1). Classification of the pathogenetic or unknown variants is explained in Supplementary Table 4.

**Fig. 2:**
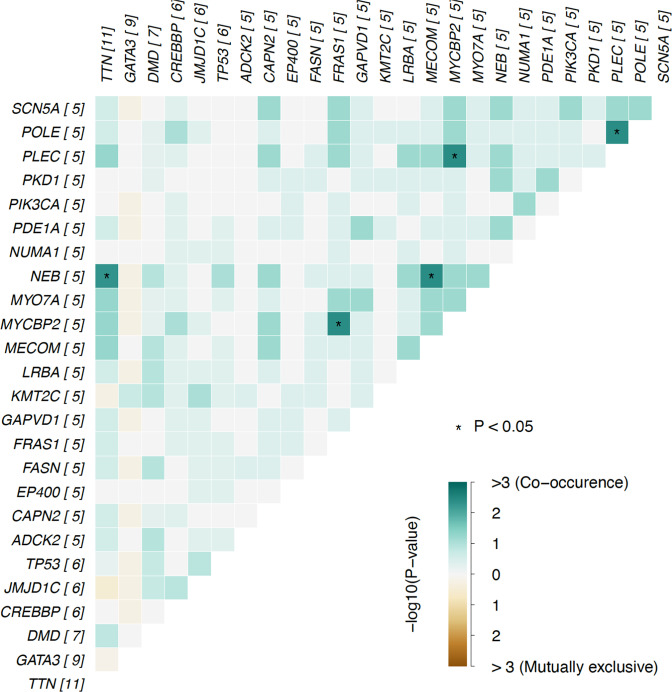
Somatic interaction analysis of the 30 most mutated genes in the breast NET cohort. The most significant co-occurring pairs of genes were MYCBP2/FRAS1 and NEB/MECOM (*p* value for both 0.004). Statistically significant pairs are indicated with darker colours and dots or asterisks if the *p* value <0.05 or < 0.01, respectively.

To investigate whether any of the most frequently mutated genes were co-occurring or mutually exclusive in the breast NET patients, somatic interaction analysis was conducted (Fig. 2). The most significant co-occurring pairs of genes were *MYCBP2/FRAS1* and *NEB/MECOM* with *p* values of 0.004. No significant mutually exclusive pairs were detected.

### Comparison between breast NET, IDC and PNET cohorts

**Fig. 3:**
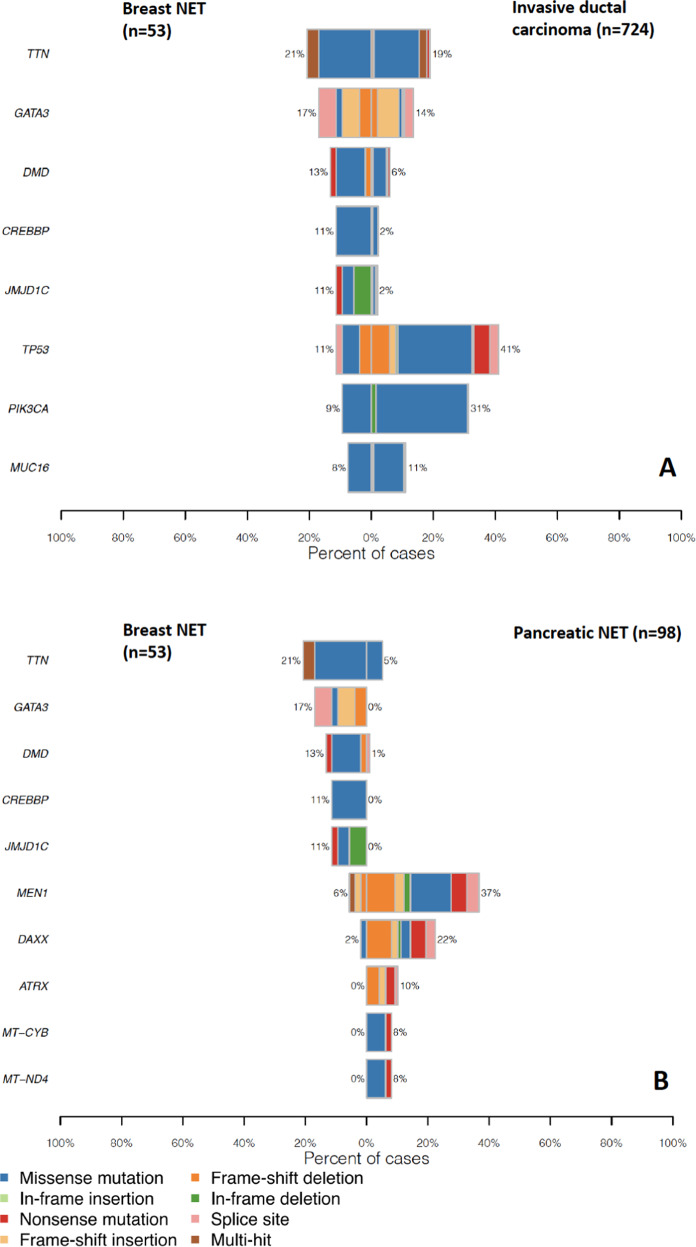
Co-bar plots comparing the top 5 most mutated genes. Co-bar plots comparing the top 5 most mutated genes from breast NET and IDC cohorts (**A**) and breast NET cohort and PNET cohorts (**B**). Especially CREBBP and JMJD1C especially were more commonly mutated in the breast NET cohort compared with the IDC cohort. Likewise, MEN1 and DAXX had rarely pathogenetic or unknown variants in breast NETs, with pathogenetic or unknown variant rates of 37% and 22% in PNETs. GATA3, CREBBP and JMJD1C pathogenetic or unknown variants were not found in PNETs, but they had an 11–17% frequency in breast NETs. TTN variants are likely not associated with cancer.

From the most mutated genes, *CREBBP* and *JMJD1C* especially were more commonly mutated in the breast NET cohort compared with the IDC cohort (Fig. 3A). Likewise, *MEN1* (6%) and *DAXX* (2%) had rarely pathogenetic or unknown variants in breast NETs, with pathogenetic or unknown variant rates of 37% and 22% in PNETs (Fig. 3B). *GATA3*, *CREBBP* and *JMJD1C* pathogenetic or unknown variants were not found in PNETs, but they had an 11–17% frequency in breast NETs. *TTN* variants are likely not associated with cancer [32]. The results indicated that the *MEN1* gene had the most significantly different pathogenetic or unknown variant rates in the patients of the two cohorts, being mutated in 36 out of 98 (36.7%) PNET samples in comparison to only 3 out of 53 (5.7%) breast NET samples (adjusted *p* value 0.00050). Altogether, 30 genes had statistically significantly different pathogenetic or unknown variant frequencies between breast NETs and PNETs after *p* value adjustment (Supplementary Fig. 2). Between breast NETs and IDCs, only pathogenetic or unknown variant frequencies of *TP53* (11.3% in breast NETs and 41% in IDCs, adjusted *p* value 0.027) and *ADCK2* (9.4% in breast NETs vs. 0.28% in IDCs, adjusted *p* value 0.045) were significantly different (Supplementary Fig. 3). Four of the five *ADCK2* pathogenetic or unknown variants observed in breast NETs were located in the same codon (Phe385Leu) (Supplementary Fig. 4). Variant allele frequency data from the most common genes showing higher pathogenetic or unknown variant rates in breast NET compared with IDC and PNET cohorts (*ADCK2, TP53, GATA3, CREBBP* and *JMJD1C*) are reported in the Supplementary Table 5. To summarise, there were substantial differences in the mutational profile of breast NET, IDC and PNET. This was exemplified by a rare occurrence of *TP53*, *PIK3CA*, *MEN1* and *DAXX* in breast NETs and a relatively high proportion of *ADCK2* pathogenetic or unknown variants.

### Oncogenic signalling pathways

**Fig. 4:**
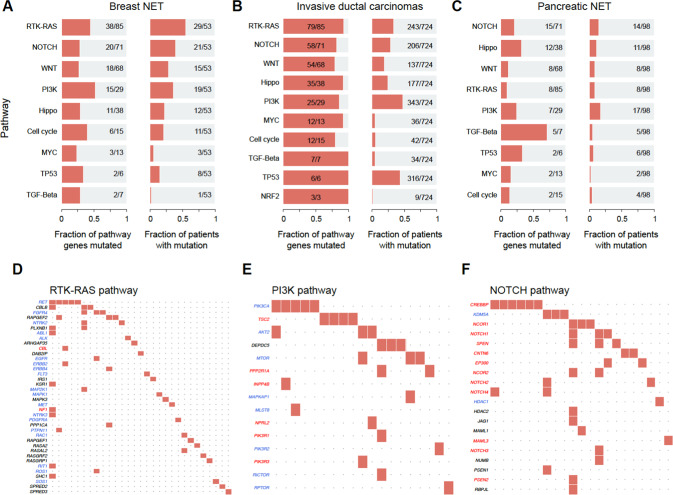
Enriched oncogenic signalling pathways in breast NETs, invasive ductal carcinomas (IDCs) and pancreatic NETs (PNETs). The bar plots on the left side in panels (**A**–**C**) show the number of mutated genes in the pathway in each of the cohorts, and the bar plots on the right side in panels (**A**–**C**) show the fractions of samples having mutated genes in the pathway. Oncoplot panels (**D**–**F**) visualise the pathogenetic or unknown variants of RTK-RAS, PI3K and NOTCH pathways in breast NETs. Tumour suppressor genes are in red font and oncogenes are in blue font in panels D–F. As the main results, the most enriched oncogenic pathways in breast NET were PI3K and RTK-RAS. A very frequent TP53 pathway enrichment in IDCs was only rarely observed in breast NETs. In PNET cohort, none of the oncogenic pathway was prominently affected over others.

Enrichment of breast NET mutated genes in known oncogenic signalling pathways (*n* = 31) were studied next. The most enriched oncogenic pathway in breast NET was PI3K (51%) and then RTK-RAS (45%) and this enrichment was found in 36% and 55% of samples for PI3K and RTK-RAS, respectively (Fig. 4A). Also in IDCs, RTK-RAS and PI3K pathways were commonly enriched (Fig. 4B). However, while there was a very frequent TP53 pathway enrichment in IDCs, this was only rarely observed in breast NETs. In PNET cohort, none of the oncogenic pathways was prominently affected by others (Fig. 4C).

### Associations between clinical and pathological parameters and pathogenetic or unknown variants in breast NETs

After multiple testing correction, higher age at diagnosis was significantly associated with the presence of pathogenetic or unknown variants in 19 genes, and pathogenetic or unknown variants in 41 genes were significantly associated with lower age (Supplementary data). Tumour size, nodal status, the presence of distant metastases at diagnosis, tumour multifocality, ER or PR expression, Ki-67 expression, HER2 amplification, immunohistochemical synaptophysin or chromogranin expression, primary tumour size (in mm), or parity were not associated with the presence of any pathogenetic or unknown variants. The association of the pathogenetic or unknown variant status with patient survival of each of the 3112 mutated genes in the filtered variant set was studied by grouping samples based on the pathogenetic or unknown variant status of each gene and performing overall and disease-specific survival analysis. None of the pathogenetic or unknown variants was statistically significantly associated with survival after multiple testing correction was applied.

### Mutational signature analysis

The de novo signature extraction with SigProfiler resulted in two SBS signatures and two ID signatures in the breast NET cohort (Supplementary Figs. 5–8, respectively). The two SBS signatures were decomposed into three COSMIC SBS signatures each, comprising four unique signatures in total (Supplementary Figs. 5–7). Two de novo ID83 signatures were found in breast NET samples. The other one of these was found to be composed of three COSMIC ID83 signatures, but the other one did not show high similarity to any of the COSMIC ID83 signatures (cosine similarity = 0.559; Supplementary Figs. 8–11).

The signatures associated with deficient DNA repair mechanisms, such as SBS6, SBS30 and ID7, were frequently found among breast NETs. Three novel signatures were found, and one of these, ID83A, was detected in 68% of breast NET cases. The results of mutational signatures are not, however, directly comparable between the cohorts, since the probability of finding a specific signature is related to the cohort size.

To summarise, the signatures SBS1 and SBS5 were the most common mutational signatures in the breast NET cohort (Table 3 and Supplementary Fig. 7). While the SBS1 signature has been linked to spontaneous deamination of 5-methylcytosine, the aetiology of SBS5 is still unknown [33].

**Table 3:** Summary table of the mutational signature analysis.

Signature	Breast NET		IDC		PNET		Proposed aetiology
	*n*	%	*n*	%	*n*	%	
SBS1	49	92	683	94	73	75	Spontaneous deamination of 5-methylcytosine (clock-like signature)
SBS2	0	0	241	33	0	0	Activity of APOBEC family of cytidine deaminases
SBS3	0	0	217	30	0	0	Defective homologous recombination DNA damage repair
SBS5	53	100	580	80	89	92	Unknown (clock-like signature)
SBS6	15	28	156	22	0	0	Defective DNA mismatch repair
SBS10a	0	0	39	5	0	0	Polymerase epsilon exonuclease domain mutations
SBS10b	0	0	133	18	0	0	Polymerase epsilon exonuclease domain mutations
SBS13	0	0	226	31	0	0	Activity of APOBEC family of cytidine deaminases
SBS29	0	0	41	6	0	0	Tobacco chewing
SBS30	8	15	0	0	18	19	Defective DNA base excision repair due to NTHL1 mutations
SBS36	0	0	0	0	6	6	Defective DNA base excision repair due to MUTYH mutations
ID1	4	8	72	10	0	0	Slippage during DNA replication of the replicated DNA strand (enriched in cancers with DNA mismatch repair deficiency)
ID2	4	8	22	3	0	0	Slippage during DNA replication of the replicated DNA strand (enriched in cancers with DNA mismatch repair deficiency)
ID3	0	0	0	0	31	32	Tobacco smoking
ID7	12	23	34	5	0	0	Defective DNA mismatch repair
ID8	0	0	301	42	0	0	Repair of DNA double-strand breaks by non-homologous end-joining mechanisms or mutations in topoisomerase TOP2A
ID11	0	0	0	0	19	20	Unknown
ID13	0	0	0	0	11	11	Ultraviolet light exposure
ID83A (breast NET novel)	36	68	0	0	0	0	?
ID83B (IDC novel)	0	0	142	20	0	0	?
ID83C (IDC novel)	0	0	314	43	0	0	?

For each COSMIC Single Base Substitution (SBS) and Insertion/Deletion (ID) signature detected, the number of samples with the signature, and the fraction of those samples in the respective cohort, are indicated.

*IDC* invasive ductal carcinoma, *PNET* pancreatic neuroendocrine tumour.

### ADCK2 mRNA and protein levels

After observing that *ADCK2* pathogenetic or unknown variant rates were substantially different between breast NET and IDC, we determined also the mRNA and protein levels of ADCK2 in the breast NET samples (Supplementary Fig. 12). ADCK2 mRNA and protein levels (with immunohistochemistry) were not different between *ADCK2*-mutated and non-mutated cases (Supplementary Figs. 13 and 14). We tested if *ADCK2* pathogenetic or unknown variants or ADCK2 mRNA or protein expression would have associations with tumour size, nodal status, the presence of distant metastases at diagnosis, tumour multifocality, ER or PR expression, Ki-67 expression, HER2 amplification, immunohistochemical synaptophysin or chromogranin expression, primary tumour size (in mm), or parity. After these analyses, only an inverse association of tumour size (T1 vs. T2–4) and ADCK2 mRNA expression (*p* = 0.040) remained statistically significant. The characteristics of the patients with ADCK2 pathogenetic or unknown variants are shown in Supplementary Table 6.

## Discussion

We studied the expression of 1099 genes from 53 breast NET samples and compared the results with pathogenetic or unknown variants from IDC and PNET. The results presented here suggest that the breast NET mutational profile is different from that of IDC, but still shares more similarities with the profile of IDCs than that of PNETs. These results also revealed several novel potentially druggable targets in breast NETs, such as *CREBBP* and MMRd.

Our study is by far the most comprehensive sequencing study performed on breast NET of 53 patients. In the pioneering study of Ang et al., the authors searched for point pathogenetic or unknown variants in 90 genes from 18 neuroendocrine breast cancers as per WHO 2003 criteria [34]. Later, Lavigne et al. checked the hotspots of 22 genes in a series of 42 neuroendocrine breast cancers [9]. More recently, two separate studies from the same group have reported breast NET mutational profiles in 10–18 samples, describing 254 of the most frequently mutated genes related to breast cancer or DNA repair [8, 10].

The main differences detected here were the low frequency of *TP53* pathogenetic or unknown variants and the high frequency of *ADCK2* pathogenetic or unknown variants in breast NETs. To the best of our knowledge, *ADCK2* pathogenetic or unknown variants have not been previously assessed in the cancer literature. Furthermore, typical pathogenetic or unknown variants of PNETs and other NENs, such as *ATRX*, *DAXX* and *MEN1*, were very rarely detected in breast NETs. *TP53* was the most commonly mutated oncogene in IDCs, being mutated in 41% of cases in the TCGA data [11]. In our PNET comparison cohort, *TP53* was mutated in only 3.1% of cases [12]. In our breast NET samples, *TP53* gene pathogenetic or unknown variants were detected in 11.3% of samples, while previous breast NET studies reported the *TP53* pathogenetic or unknown variants frequency to be between 0 and 7% [9, 10]. The mutational spectrum of *TP53* in our material was otherwise in line with the TCGA IDC data, but no nonsense *TP53* pathogenetic or unknown variants were observed in our patients. *PIK3CA* is another of the most commonly mutated genes in IDCs, especially in the luminal A subtype, where most breast NETs also fit [11]. In our breast NET cohort, only 9% of tumours harboured *PIK3CA* pathogenetic or unknown variants, while the rate was 31% in the TCGA IDC material. Previously, *PIK3CA* pathogenetic or unknown variants have been reported in breast NETs in 7–20% of cases, in studies with smaller sample sizes and more limited sequencing [8, 9]. Nevertheless, according to our analysis, the *PI3K*/*Akt*/*mTOR* pathway, *NOTCH* pathway and *RTK-RAS* pathway were the most commonly affected oncogenic pathways in breast NETs. From the therapeutic point of view, all three of these pathways are well druggable with US Food and Drug Administration (FDA)-approved compounds [35–37]. Thus, also in terms of affected pathways, breast NETs are more comparable to IDCs than PNETs, with the transforming growth factor beta pathway demonstrating the most pathogenetic or unknown variants [11, 12]. The transcription factor GATA binding protein 3 (*GATA3*), responsible for mammary gland development and for luminal transcription programme, was the most frequently mutated gene in breast NETs. The *GATA3* pathogenetic or unknown variant rate was comparable to that of the IDC cohort (17 and 14%, respectively), whereas no *GATA3* pathogenetic or unknown variants were detected in our PNET cohort. In agreement with this, the Reis-Filho group reported *GATA3* pathogenetic or unknown variants in one of their 10 breast NET samples [8]. GATA3 protein expression and *GATA3* pathogenetic or unknown variants may predict better prognosis, at least in ER-positive breast cancers [38, 39]. Nevertheless, the exact biological significance of *GATA3* pathogenetic or unknown variants remains unclear [40]. IDC-related *GATA3* pathogenetic or unknown variants are mainly concentrated in exons 5 and 6 encoding for the C-terminal region of the protein, and are almost solely frame shift pathogenetic or unknown variants, which is concordant with pathogenetic or unknown variants observed in our breast NET patients [41]. ADCK2 is a mitochondrial protein kinase whose function is still poorly understood. However, other members of the ADCK family have been implicated in ubiquinone biosynthesis [42]. According to the publicly available data from TCGA, the prevalence of *ADCK2* pathogenetic or unknown variants in most cancers is 0%, rarely above 1%, with the maximum pathogenetic or unknown variant frequencies observed in endometrial carcinomas and in rare uterine carcinosarcomas, up to 3.5% of cases [43, 44]. *ADCK2* pathogenetic or unknown variants were extremely rare in IDCs, 0.28%, and no *ADCK2* pathogenetic or unknown variants were found in the PNET cohort [11, 12]. In breast NETs, we found *ADCK2* missense pathogenetic or unknown variants in 5/53 (9.4%) cases, four of them being located in the same codon (Phe385Leu). Interestingly, *ADCK2* inhibition abrogates oestrogen-related signalling, including ESR1 in ER-positive breast cancers [45]. ADCK2 protein expression has been reported to be elevated in luminal A compared to luminal B breast cancers [46]. Lately, high ADCK2 protein expression has been proposed to be a marker for an improved therapeutic response during breast cancer neoadjuvant treatment [47]. The observed *ADCK2* pathogenetic or unknown variants did not, however, associate with mRNA or protein expression. The number of cases here was, however, too small for any conclusions about this. Furthermore, possible changes caused by the detected pathogenetic or unknown variants on putative enzyme activity or substrate specificity cannot be ruled out. Nevertheless, the suggested inverse association between ADCK2 mRNA levels and tumour size encourages further investigation into its role in tumour development. Pathogenetic or unknown variants in some central epigenetic regulators were overrepresented in breast NETs compared to IDCs. Lysine acetyltransferase *CREBBP* was mutated in 11% of breast NETs, which is of a similar magnitude to that reported previously in neuroendocrine small-cell lung cancer [48]. TCGA material reported a pathogenetic or unknown variant rate of only 2% for IDCs, but in rare adenoid cystic carcinomas of the breast, 31% of tumours have shown *CREBBP* pathogenetic or unknown variants [12, 49]. Interestingly, there is recent evidence from multiple tumour types that histone deacetylase inhibitors could offer exceptionally high response rates in tumours harbouring *CREBBP* pathogenetic or unknown variants [48, 50]. Likewise, *JMJD1C*, a histone demethylase associated with epigenetic regulation, harboured pathogenetic or unknown variants in 11% of our breast NETs and only 2% in IDCs.

In contrast to the rather comparable mutational profile between breast NETs and IDCs, breast NETs and PNETs demonstrated more major differences. *MEN1* is a tumour suppressor gene participating in DNA stability and gene regulation. Germline *MEN1* pathogenetic or unknown variants, with a prevalence of 3–4/100,000 in the Caucasian population, predispose to duodenopancreatic NETs [51]. In PNETs, pathogenetic or unknown variants in the *MEN1* gene have been reported in 37–44% of cases [12, 52]; whereas in TCGA IDC material, only 0.9% of cases harboured *MEN1* pathogenetic or unknown variants [11]. Thus, our breast NET material, with 6% of patients carrying *MEN1* pathogenetic or unknown variants in their tumours, seems to be an intermediate form between IDCs and PNETs. Interestingly, in our recent study, low MEN1 protein expression was associated with poor local relapse-free survival in breast NETs [4]. Whether the low MEN1 expression was actually due to the pathogenetic or unknown variant was not, however, addressed [53]. Pathogenetic or unknown variants in chromatin remodelling genes *DAXX* and *ATRX* are also characteristic of PNETs, and they are also frequent in other NENs [13, 54]. Similarly with MEN1, these pathogenetic or unknown variants were still very rare in breast NETs: none of the samples harboured *ATRX* pathogenetic or unknown variants, and only one patient had a *DAXX* pathogenetic or unknown variants. Previously *ATRX* pathogenetic or unknown variants have been reported in 0–0.9% of breast cancers, depending on the biological subtype [55, 56]. There are no previous reports on *DAXX* pathogenetic or unknown variants in breast cancer.

Breast cancer mutational signatures have been recently described [57]. They provide insight into carcinogenetic processes and also have the potential to influence cancer diagnostics and treatment [58]. In breast NETs, the most common mutational signatures were related to aging (SBS1 and SBS5), a finding which is in line with the generally older age of the population with this disease. Interestingly, the SBS6 and ID7 signatures associated with defective mismatch repair (MMRd) were recorded in 28% and 23% of cases, while they were quite rare in IDCs and absent in PNETs. MMRd tumours are sensitive to PD-1 inhibitors, and the FDA has recently approved pembrolizumab for tumour-agnostic treatment of MMRd solid tumours. Thus, a subset of breast NET patients might benefit from MMRd testing. Finally, our analysis and previous data suggest the presence of APOBEC-related mutational signatures SBS2 and SBS13 in one-third of the IDC patients. In breast NET cases, these signatures were not found [57].

We acknowledge several pitfalls in our study design. First, non-cancerous tissue was not available from the same patients. We were thus unable to make definite divisions between somatic and germline pathogenetic or unknown variants and e.g. copy number alteration analyses were not possible. We took, however, various approaches to enrich somatic pathogenetic or unknown variants, such as the exclusion of any pathogenetic or unknown variant rate higher than 1% in public databases as well as any pathogenetic or unknown variants with non-high degree of pathogeny. We are aware that this filtering may have resulted in the exclusion of some somatic pathogenetic or unknown variants. Our study may also be criticised for using TCGA data for all IDC cases and for not selecting only ER-positive and HER2-negative tumours. Although the majority of breast NETs showed a luminal A-like phenotype, there were also ER-negative and HER2-positive breast NETs, as also demonstrated by breast NET cases in this study. PNETs were chosen as a NET comparator group as these tumours are among the most deeply characterised malignant NETs. On the other hand, e.g., neuroendocrine lung cancers have a much more aggressive clinical course compared to breast NETs.

We conclude that the breast NET mutational profile, including oncogenic pathway analysis, more closely resembles that of IDCs than PNETs. There are, however, certain differences between breast NET and IDC mutational profiles, as exemplified by those in the *MEN1* and *ADCK2* genes. Taken together, these findings suggest that breast NETs are indeed a separate breast cancer entity. Whether it should be treated differently, e.g. according to their mutational signature profiles, remains to be elucidated. This study may lay the foundation for future studies evaluating potential therapeutic breast NET targets, such as *CREBBP*.

## Supplementary information

Supplementary data

## Data Availability

Data are available upon reasonable request.
