# Supplementary material for: Comparison of the mutational profiles of neuroendocrine breast tumours, invasive ductal carcinomas and pancreatic neuroendocrine carcinomas
Source: Oncogenesis. 2022 Sep 9;11(1):53. doi: 10.1038/s41389-022-00427-1 (PMC9463436; doi:10.1038/s41389-022-00427-1)
Supplement: Supplementary file 1 — Supplementary data [file 41389_2022_427_MOESM1_ESM.docx]

**Supplementary data**

**Methods**

*ADCK2 mRNA analysis and immunostaining*

Total RNA was extracted from formalin-fixed, paraffin-embedded (FFPE) breast tissue sections using the miRNeasy FFPE kit and the QIAcube instrument according to the manufacturer’s protocol (Qiagen, Hilden, Germany). The miScript II Reverse Transcription Kit (Qiagen) was used for synthesis of cDNAs. Amplifications were made with a Rotor-Gene Q instrument (Qiagen) and Luna® Universal qPCR Master Mix (New England Biolabs, MA, USA) using primers F 5’- CCCCTTTGTCACCAGAGAAGT -3’, R 5’- CTTCCTTTTCAAGTCCACGGG -3’ for *ADCK2* (NM052853.4) and F: 5’-TGGAAGGACTCATGACCACA-3’ and R: 5’-CCATCACGCCACAGTTT-3’ for the *GAPDH* (BC029618) reference gene. The PCR protocol consisted of a denaturation cycle (+95°C 1 min) followed by 40 cycles for two-step amplification (+95°C 15 s, +60°C 30 s). The fold change in relative expression of *ADCK2* was calculated using the 2^-ΔΔCt^ method (33).

The paraffin-embedded breast tissue specimens were cut into 3.5-mm sections, mounted on silane-coated slides and incubated at 37^o^C overnight. The sections were then processed using BOND RX automate and the BOND Polymer Refine Detection kit (Leica Biosystems Ltd, Newcastle, UK). The procedure included deparaffinization and heat-induced epitope retrieval (ER1 citrate pH 6 buffer at 100^o^C for 30 min). Sections were incubated for 3 hours at ambient temperature with 1:20 diluted ADCK2 antibody (Novus Biologicals, NBP1-82736) and counterstained with hematoxylin. Human kidney FFPE tissue was used as a positive control. PBS buffer was used instead of the antibody in the negative control. Two authors (PK and KMH) performed the evaluation of immunostaining for tumour cell cytoplasm and nuclei. The intensity of the staining of each cell compartment was evaluated as 0 (negative), 1 (weakly positive), 2 (moderately positive) or 3 (strongly positive). The number of stained cells was reported as percentages (0–100) out of all malignant cells. A histological sum score, the H score, was computed by multiplying the intensity and staining percentage scores, resulting in a scale of 0–300. It was tested if *ADCK2* pathogenetic or unknown variants or ADCK2 mRNA or protein expression would have associations with tumour size, nodal status, the presence of distant metastases at diagnosis, tumour multifocality, ER or PR expression, Ki-67 expression, HER2 amplification, immunohistochemical synaptophysin or chromogranin expression, primary tumour size (in mm), or parity.

*Mutational signature analysis*

The analysis included the extraction of adjacent bases surrounding the mutated bases and the generation of a pathogenetic or unknown variant matrix that classified nucleotide substitutions into 96 substitution classes based on the surrounding bases using the *trinucleotideMatrix* function. The number of signatures was then estimated by non-negative matrix factorisation (NMF) using the *estimateSignatures* function. The optimal number of signatures – three – was determined based on the cophenetic correlation metric. The pathogenetic or unknown variant matrix was then decomposed into three signatures using the *extractSignatures* function. The extracted signatures were compared to previously characterised COSMIC signatures, both 30 legacy signatures and a more recent set of 65 Single Base Substitution (SBS) signatures, using the *compareSignatures* function. The obtained signatures were visualised using the *plotSignatures* function. In addition, the *plotApobecdiff* function was used for analysing the differences in mutational patterns between APOBEC-enriched and non-APOBEC-enriched samples using the APOBEC enrichment scores estimated by the *trinucleotideMatrix* function for the APOBEC enrichment classification of the samples.

To more comprehensively analyse the mutational signatures of the filtered breast NET cohort variants as well as the PNET and BRCA-IDC variants, SigProfiler software (Bergstöm et al., 2019) v. 3.1, was used. First, the *SigProfilerMatrixGenerator* tool was used for creating mutational matrices, categorising the mutations based on possible single nucleotide variants (SNVs; SBS-6, SBS-24, SBS-96, SBS-384, SBS-1536 and SBS-6144 categories), double base substitutions (DBS; DBS-78, DBS-186, DBS-1248 and DBS-2976 categories) and insertions/deletions (ID; ID-28, ID-83, ID-415 and ID-8268 categories). The *SigProfilerExtractor* tool was then used for *de novo* extraction of mutational signatures from the mutational matrices of the most commonly used SBS-96 classification, DBS-78 classification and ID-83 classification of SNVs, DBSs and IDs, respectively. Based on the mean sample cosine distance and the average stability, the tool selected the optimal number of signatures and provided *de novo* signature extraction results as well as COSMIC signature decomposed solution results for these signatures. The tool computed the activity of each signature in each sample, the tumour mutational burden (TMB) of the samples containing the mutational signatures, and the contributions of each pathogenetic or unknown variant to the observed signature. For the COSMIC signature decomposed solution, the tool provided information on the COSMIC signatures of which the *de novo* extracted signatures were composed and on the reconstruction of the *de novo* signatures based on the COSMIC signature decomposition.

Supplementary Table 1. MuTect2 metrics and filters used to exclude artefacts from the variant calls.

| Metric/Filter | Description |
| --- | --- |
| Multiallelic | Multiple alleles are found at a given site. This often indicates alignment issues since it is unlikely that somatic pathogenetic or unknown variants occur at the same exact site. |
| Contamination | Observed cross contamination. |
| Mapping quality | The median mapping quality for the reads supporting the alternative (variant) allele is low. |
| Base quality | The median base quality for the base calls representing the alternative (variant) allele is low. |
| Read position | The median distance of the pathogenetic or unknown variant position from the end of the reads supporting the alternative allele is low. Often, pathogenetic or unknown variants occurring at the ends of reads are false positives because of the low overall quality of the 3’ends of reads. |
| Clustered events | The number of pathogenetic or unknown variant events in a local assembly site is too high. GATK performs a local realignment procedure to improve the alignment at sites of indels. Local assembly refers to this region. Similar to multiallelic site somatic pathogenetic or unknown variants. |
| Fragment length | The median difference between fragments (estimated based on read pairs) that support reference and alternative alleles is too high. |
| Strand artifact | The presentation of pathogenetic or unknown variants in terms of strands is too biased towards one of the two possible strands. |
| Bad haplotype | Pathogenetic or unknown variants occurs in the same haplotype as a filtered low-quality pathogenetic or unknown variant. |
| Str contraction | Indel occurs at a satellite repeat region. Indels that occur within satellite repeats are likely false positives, as it is challenging to correctly call pathogenetic or unknown variants in these regions. |
| Panel of normals | Pathogenetic or unknown variants is blacklisted in the panel of normals. In other words, it is considered a recurring sequencing artefact. |

Supplementary Table 2. Population databases included in Variant Effect Predictor.

| Database/subset | Description |
| --- | --- |
| 1000 Genomes | Frequency of existing variant in 1000 Genomes combined population |
| 1000 Genomes AFR | Frequency of existing variant in 1000 Genomes combined African population |
| 1000 Genomes subset AMR | Frequency of existing variant in 1000 Genomes combined American population |
| 1000 Genomes subset EAS | Frequency of existing variant in 1000 Genomes combined East Asian population |
| 1000 Genomes subset EUR | Frequency of existing variant in 1000 Genomes combined European population |
| 1000 Genomes subset SAS | Frequency of existing variant in 1000 Genomes combined South Asian population |
| NHLBI-ESP AA | Frequency of existing variant in NHLBI-ESP African American population |
| NHLBI-ESP EA | Frequency of existing variant in NHLBI-ESP European American population |
| gnomAD AF | Frequency of existing variant in gnomAD exomes combined population |
| gnomAD AFR | Frequency of existing variant in gnomAD exomes African American population |
| gnomAD AMR | Frequency of existing variant in gnomAD exomes American population |
| gnomAD ASJ | Frequency of existing variant in gnomAD exomes Ashkenazi Jewish population |
| gnomAD EAS | Frequency of existing variant in gnomAD exomes East Asian population |
| gnomAD FIN | Frequency of existing variant in gnomAD exomes Finnish population |
| gnomAD NFE | Frequency of existing variant in gnomAD exomes non-Finnish European population |
| gnomAD OTH | Frequency of existing variant in gnomAD exomes other combined populations |
| gnomAD SAS | Frequency of existing variant in gnomAD exomes South Asian population |

Supplementary Table 3. Additional filtering methods attempted for the breast NET variant set.

| Filtered variant set | N of variants before filtering | N of variants after filtering | Filtering criteria | Notes |
| --- | --- | --- | --- | --- |
| 1 | 16264 | 3869 | Variant kept if ‘Damaging’ prediction by either DEOGEN2 or primateAI | Many variants without annotation (including all frame-shift indels) excluded |
| 2 | 16264 | 8162 | Variant excluded if ‘Tolerated’ prediction by both DEOGEN2 and primateAI | Several likely germline variants still left in the set |
| 3 | 8162 | 1432 | Set 2 filtered further by excluding all variants annotated as germline risk variants by Mutect2 | May be too conservative as the germline risk prediction was not very accurate due to a lack of matched normal tissue samples |
| 4 | 16264 | 14990 | Variant kept if either Gnomad_exome_AF or Gnomad_exome_FIN_AF <0.2% (or not known) | Only small portion of variants filtered out |
| 5 | 14990 | 2390 | Set 4 filtered further by excluding variants annotated as germline risk variants by Mutect2 | Still contained many variants that were more likely to be germline than somatic as they were present in multiple samples |
| 6 | 16264 | 2376 | Variant kept if either Gnomad_exome_AF or Gnomad_exome_FIN_AF <0.1% (or not known) and not annotated as germline risk variant by Mutect2 | Very similar outcome as in Set 4 |
| 7 | 2390 | 1428 | Set 5 filtered further by excluding variants with ‘Tolerated’ prediction by both DEOGEN2 and primateAI | Still contained variants that were more likely to be germline than somatic as they were present in multiple samples |
| 8 | 16264 | 4360 | Missense variants kept if CHASM score > 0.5, other variant types kept if VEST score > 0.5 | Appeared to work better for filtering based on pathogenicity than DEOGEN2 and primateAI as most variants had pathogenicity scores that could be used |
| 9 | 16264 | 7171 | Missense variants kept if CHASM score or VEST score > 0.5, other variant types kept if VEST score > 0.5 | Could be used but using only CHASM scores for the missense variants may produce more relevant pathogenetic or unknown variants for cancer samples |
| 10 | 7171 | 1126 | Set 9 variants were filtered further by excluding variants annotated as germline risk variants by Mutect2 and variants found in more than 5 samples | May be too conservative as the germline risk prediction was not very accurate due to a lack of matched normal tissue samples |
| 11 | 4360 | 4330 | Set 8 variants were filtered further to exclude variants found in more than 5 samples | Final filtered variant set to be used in the downstream analysis |

Supplementary Table 4. Descriptions of variant classes. Adapted from Ensembl glossary (https://www.ensembl.org/Help/Glossary).

| Variant classification | Description |
| --- | --- |
| Missense mutation | A sequence variant that changes one or more bases, resulting in a different amino acid sequence but whose length is preserved |
| Splice site | A sequence variant in which a change has occurred within the region of the splice site |
| Nonsense mutation | A sequence variant whereby at least one base of a codon is changed, resulting in a premature stop codon and leading to a shortened transcript |
| Frame-shift deletion | A sequence variant that causes a disruption of the translational reading frame because the number of nucleotides deleted is not a multiple of three |
| Frame-shift insertion | A sequence variant that causes a disruption of the translational reading frame because the number of nucleotides inserted is not a multiple of three |
| In-frame deletion | An in-frame non-synonymous variant that deletes bases from the coding sequence |
| In-frame insertion | An in-frame non-synonymous variant that inserts bases from the coding sequence |
| Non-stop mutation | A sequence variant in which at least one base of the terminator codon (stop) is changed, resulting in an elongated transcript |
| Translation start site | A codon variant that changes at least one base of the canonical start codon |

Supplementary Table 5. Identified pathogenetic or unknown variants in five selected genes. Variant allele fraction is calculated as the number of reads with the alternative allele over all reads at the variant site. Note that the ADCK2 pathogenetic or unknown variant in four of the patients are identical.

| **Gene** | **Chromosome** | **Start Position** | **End Position** | **Reference Allele** | **Alternative Allele** | **Variant Type** | **Variant Classification** | **Protein position** | **Amino acids** | **Tumor Sample Barcode** | **Variant allele fraction** |  |
| --- | --- | --- | --- | --- | --- | --- | --- | --- | --- | --- | --- | --- |
|  |  |  |  |  |  |  |  |  |  |  |  |  |
| ADCK2 | chr7 | 140679229 | 140679229 | C | G | SNP | Missense | 385 | F/L | KS_Ex_20_S5 | 0,25 |  |
| ADCK2 | chr7 | 140679229 | 140679229 | C | G | SNP | Missense | 385 | F/L | KS_Ex_37_S3 | 0,2 |  |
| ADCK2 | chr7 | 140679229 | 140679229 | C | G | SNP | Missense | 385 | F/L | KS_Ex_47_S10 | 0,26 |  |
| ADCK2 | chr7 | 140679229 | 140679229 | C | G | SNP | Missense | 385 | F/L | KS_Ex_56_S13 | 0,3 |  |
| ADCK2 | chr7 | 140694747 | 140694747 | G | A | SNP | Missense | 609 | D/N | KS_Ex_39_S5 | 0,48 |  |
| CREBBP | chr16 | 3727837 | 3727837 | C | T | SNP | Missense | 2404 | E/K | KS_Ex_21_S6 | 0,32 |  |
| CREBBP | chr16 | 3727896 | 3727896 | T | G | SNP | Missense | 2384 | H/P | KS_Ex_31_S11 | 0,29 |  |
| CREBBP | chr16 | 3727942 | 3727942 | G | T | SNP | Missense | 2369 | P/T | KS_Ex_13_S11 | 0,7 |  |
| CREBBP | chr16 | 3728423 | 3728423 | T | G | SNP | Missense | 2208 | Q/H | KS_Ex_22_S7 | 0,75 |  |
| CREBBP | chr16 | 3728423 | 3728423 | T | G | SNP | Missense | 2208 | Q/H | KS_Ex_52_S2 | 0,37 |  |
| CREBBP | chr16 | 3769293 | 3769293 | C | T | SNP | Missense | 981 | A/T | KS_Ex_8_S6 | 0,38 |  |
| GATA3 | chr10 | 8058529 | 8058529 | A | C | SNP | Missense | 156 | T/P | KS_Ex_28_S8 | 0,15 |  |
| GATA3 | chr10 | 8069469 | 8069469 | TCA | T | DEL | Splice site |  |  | KS_Ex_16_S1 | 0,46 |  |
| GATA3 | chr10 | 8069469 | 8069469 | TCA | T | DEL | Splice site |  |  | KS_Ex_24_S9 | 0,27 |  |
| GATA3 | chr10 | 8069469 | 8069469 | TCA | T | DEL | Splice site |  |  | KS_Ex_42_S8 | 0,42 |  |
| GATA3 | chr10 | 8073761 | 8073761 | AGAAG | A | DEL | Frame-shift deletion | 358-359 | KK/X | KS_Ex_25_S5 | 0,59 |  |
| GATA3 | chr10 | 8073765 | 8073765 | G | GGA | INS | Frame-shift insertion | 359-360 | -/X | KS_Ex_51_S1 | 0,36 |  |
| GATA3 | chr10 | 8073990 | 8073990 | CCACCCCT | C | DEL | Frame-shift deletion | 435-437 | HPS/X | KS_Ex_6_S4 | 0,19 |  |
| GATA3 | chr10 | 8073992 | 8073992 | A | AC | INS | Frame-shift insertion | 435 | H/HX | KS_Ex_47_S10 | 0,33 |  |
| GATA3 | chr10 | 8074016 | 8074016 | T | TG | INS | Frame-shift insertion | 443 | M/MX | KS_Ex_48_S11 | 0,47 |  |
| JMJD1C | chr10 | 63206628 | 63206628 | T | A | SNP | Nonsense | 1681 | R/* | KS_Ex_12_S10 | 0,37 |  |
| JMJD1C | chr10 | 63215047 | 63215047 | C | G | SNP | Missense | 374 | D/H | KS_Ex_21_S6 | 0,55 |  |
| JMJD1C | chr10 | 63215293 | 63215293 | CCTT | C | DEL | In-frame deletion | 328 | K/- | KS_Ex_20_S5 | 0,57 |  |
| JMJD1C | chr10 | 63215293 | 63215293 | CCTT | C | DEL | In-frame deletion | 328 | K/- | KS_Ex_32_S12 | 0,56 |  |
| JMJD1C | chr10 | 63215293 | 63215293 | CCTT | C | DEL | In-frame deletion | 328 | K/- | KS_Ex_7_S5 | 0,55 |  |
| JMJD1C | chr10 | 63215319 | 63215319 | C | T | SNP | Missense | 320 | S/N | KS_Ex_13_S11 | 0,5 |  |
| TP53 | chr17 | 7673751 | 7673751 | C | T | SNP | Missense | 290 | R/H | KS_Ex_29_S9 | 0,51 |  |
| TP53 | chr17 | 7673778 | 7673778 | T | G | SNP | Missense | 281 | D/A | KS_Ex_53_S3 | 0,86 |  |
| TP53 | chr17 | 7675140 | 7675140 | G | A | SNP | Missense | 158 | R/C | KS_Ex_4_S2 | 0,55 |  |
| TP53 | chr17 | 7675238 | 7675238 | T | A | SNP | Splice site |  |  | KS_Ex_7_S5 | 0,71 |  |
| TP53 | chr17 | 7676004 | 7676004 | AC | A | DEL | Frame-shift deletion | 122 | V/X | KS_Ex_15_S13 | 0,57 |  |
| TP53 | chr17 | 7676194 | 7676194 | CTGGGTCTTCA | C | DEL | Frame-shift deletion | 55-58 | TEDP/X | KS_Ex_20_S5 | 0,86 |  |

Supplementary Table 6. Clinical and pathological characteristics of the five patients and tumours having pathogenetic or unknown variants in ADCK2 gene. *ADCK2* pathogenetic or unknown variants did not have significant associations with clinicopathological traditional prognostic parameters.

|  | Patient 1 | Patient 2 | Patient 3 | Patient 4 | Patient 5 |
| --- | --- | --- | --- | --- | --- |
| Age at diagnosis | 60 | 82 | 77 | 66 | 60 |
| Menopausal status | Postmenopausal | Postmenopausal | Postmenopausal | Postmenopausal | Postmenopausal |
| Tumour size (mm) | 25 | 20 | 20 | 20 | 16 |
| T-class | T2 | T1c | T1c | T1c | T1c |
| Axillary nodal metastases | 2 | 0 | 0 | 0 | 0 |
| Distant metastases at diagnosis | No | No | No | No | No |
| Multifocal cancer | Yes | No | No | No | No |
| ER expression | > 60% | > 60% | > 60% | > 60% | > 60% |
| PR expression | 1–9% | 10–59% | > 60% | 10–59% | > 60% |
| HER2 amplification | Negative | Negative | Negative | Negative | Negative |
| Ki-67 expression | 15–30% | > 30% | 5–14% | 5–14% | > 30% |
| Synpatophysin expression | Present | Present | Present | Present | Present |
| Chromogranin expression | Not present | Not present | Not present | Present | Not present |
| Recurrence | Yes | No | No | No | No |


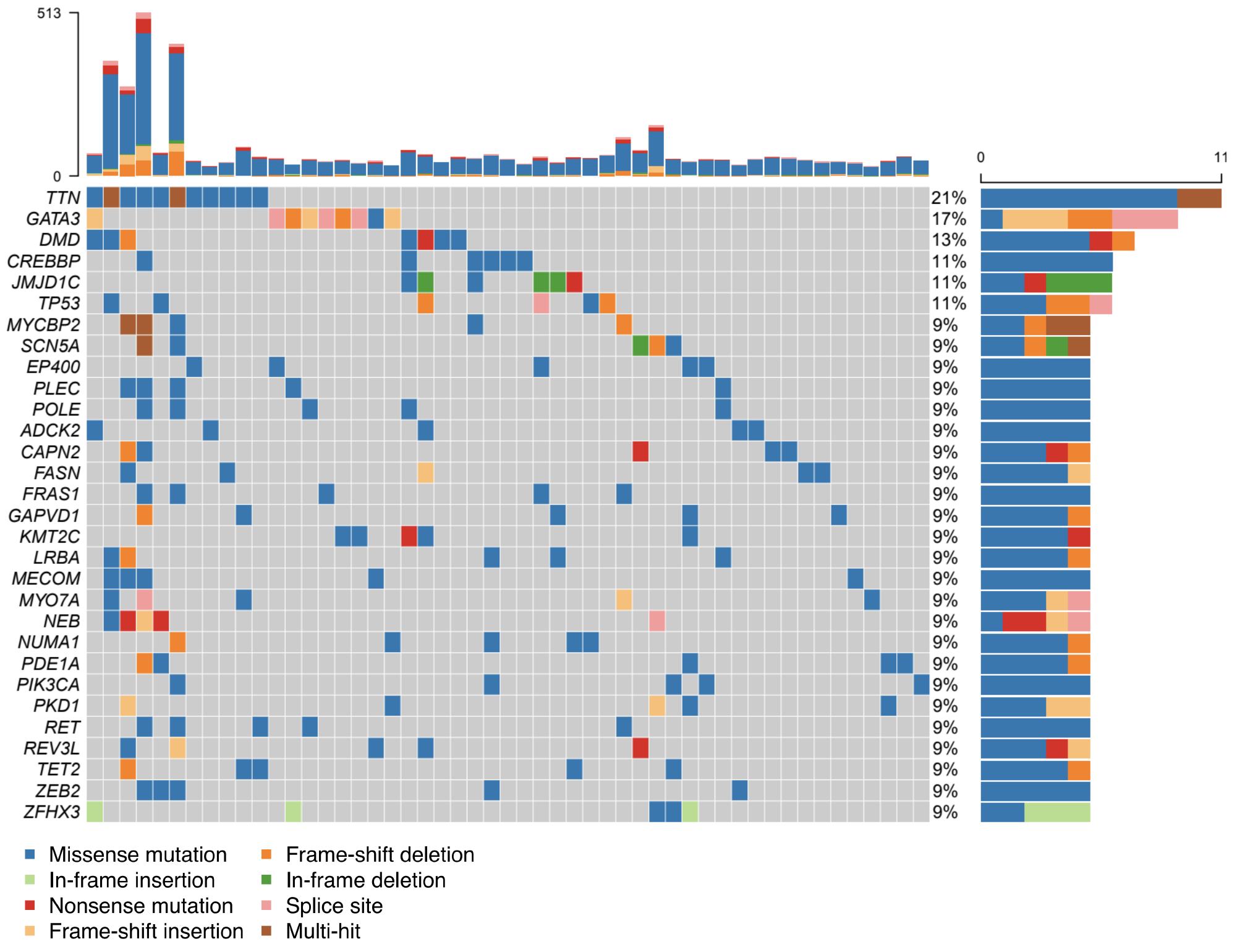


Supplementary Figure 1. An oncoplot showing the 30 most mutated genes in the breast NET cohort. TTN, GATA3, DMD, CRBBP and JMJD1C were the most frequently mutated genes with 21, 17, 13, 11 and 11 percent of all breast NET patients affected, respectively. At least TTN and PLEC pathogenetic or unknown variants are likely not associated with cancer due to their length.


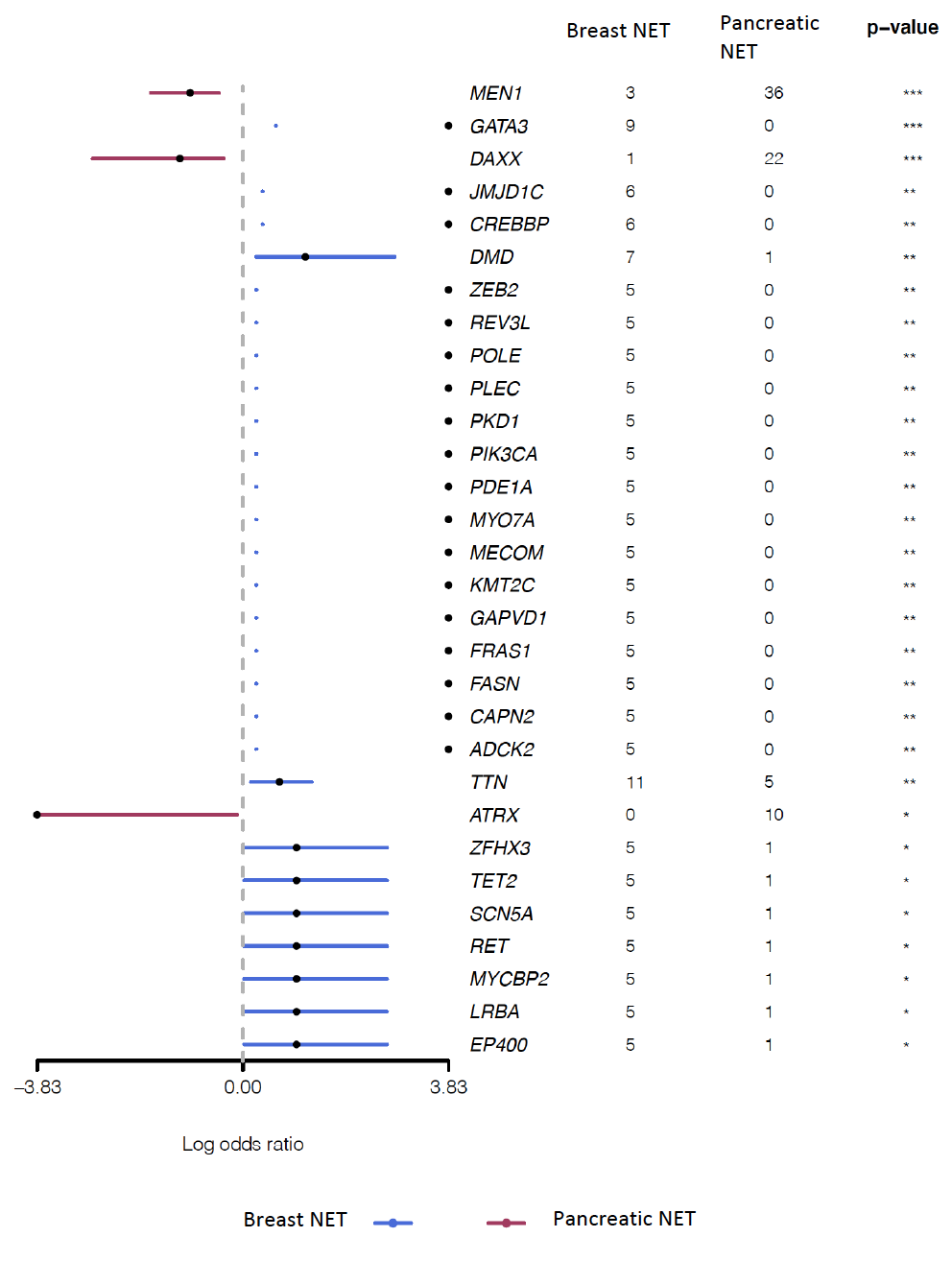


Supplementary Figure 2. A forest plot of the significantly (adjusted p-value < 0.05) differentially mutated genes in breast NETs (n=53) and pancreatic NETs (n=98). The red bars indicate higher incidence of the pathogenetic or unknown variant in PNET while the blue bars indicate higher incidence in breast NET. p-value ≤ 0.05 (*), ≤ 0.01 (**) and ≤ 0.001 (***).


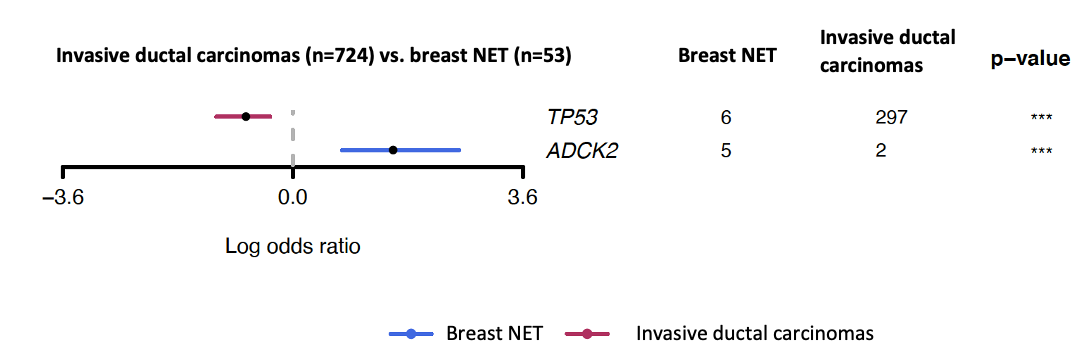


Supplementary Figure 3. A forest plot of the significantly (adjusted p-value < 0.05) differentially mutated genes in invasive ductal carcinomas (n=724) and breast NET (n=53). The red bar indicates higher incidence of the pathogenetic or unknown variant in invasive ductal carcinomas while the blue bar indicates higher incidence in breast NET. p-value ≤ 0.001 (***).


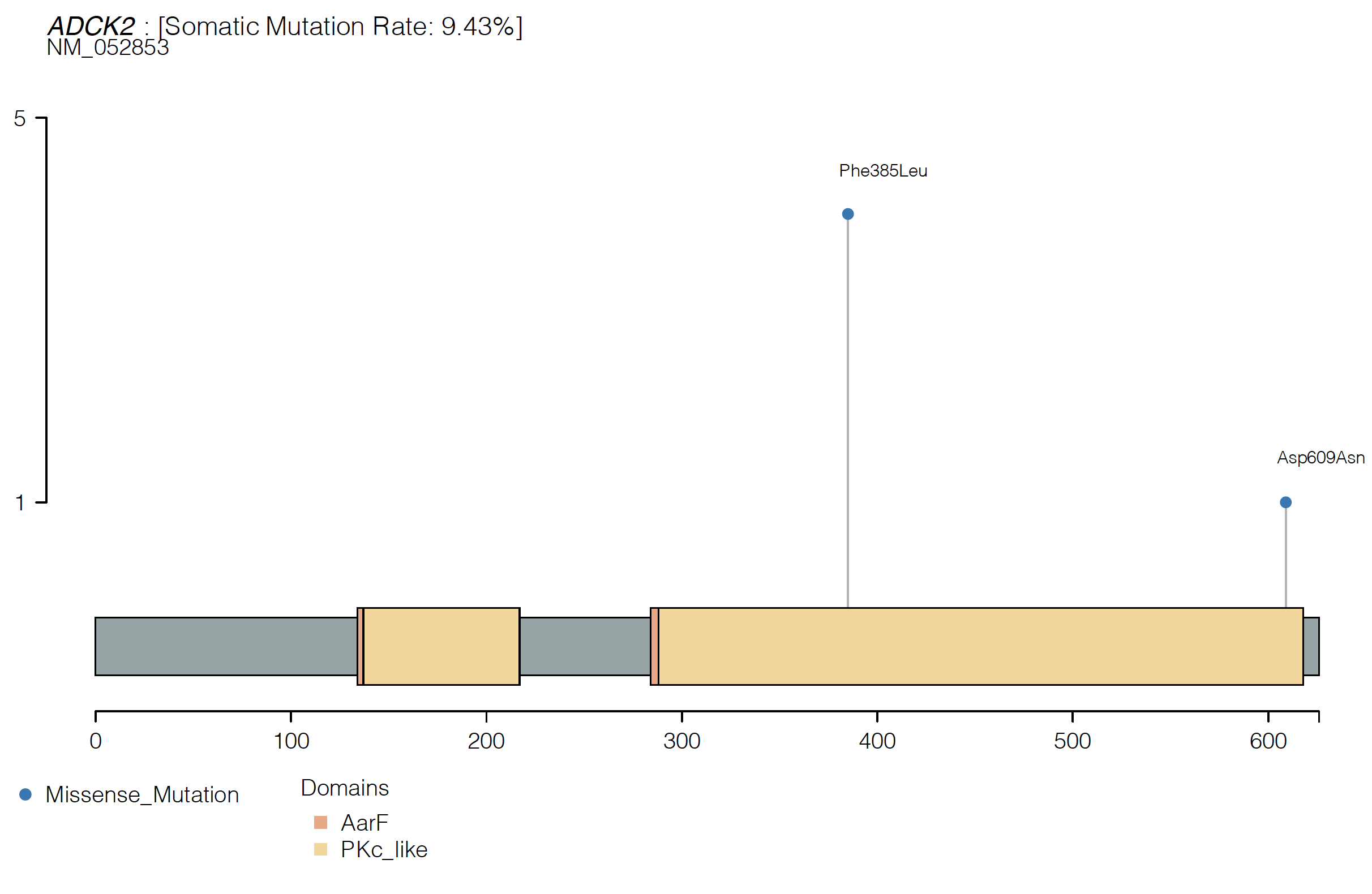


Supplementary Figure 4. Lollipop plot of ADCK2 gene, showing the amino acid changes caused by the somatic pathogenetic or unknown variants observed in the breast NET patients. Protein domain annotations obtained from the protein families database are also shown in the plot as coloured squares, with domain names indicated in the legend below the plot.

Supplementary Figure 5. Activity plot of the de novo signatures extracted with SigProfiler in breast
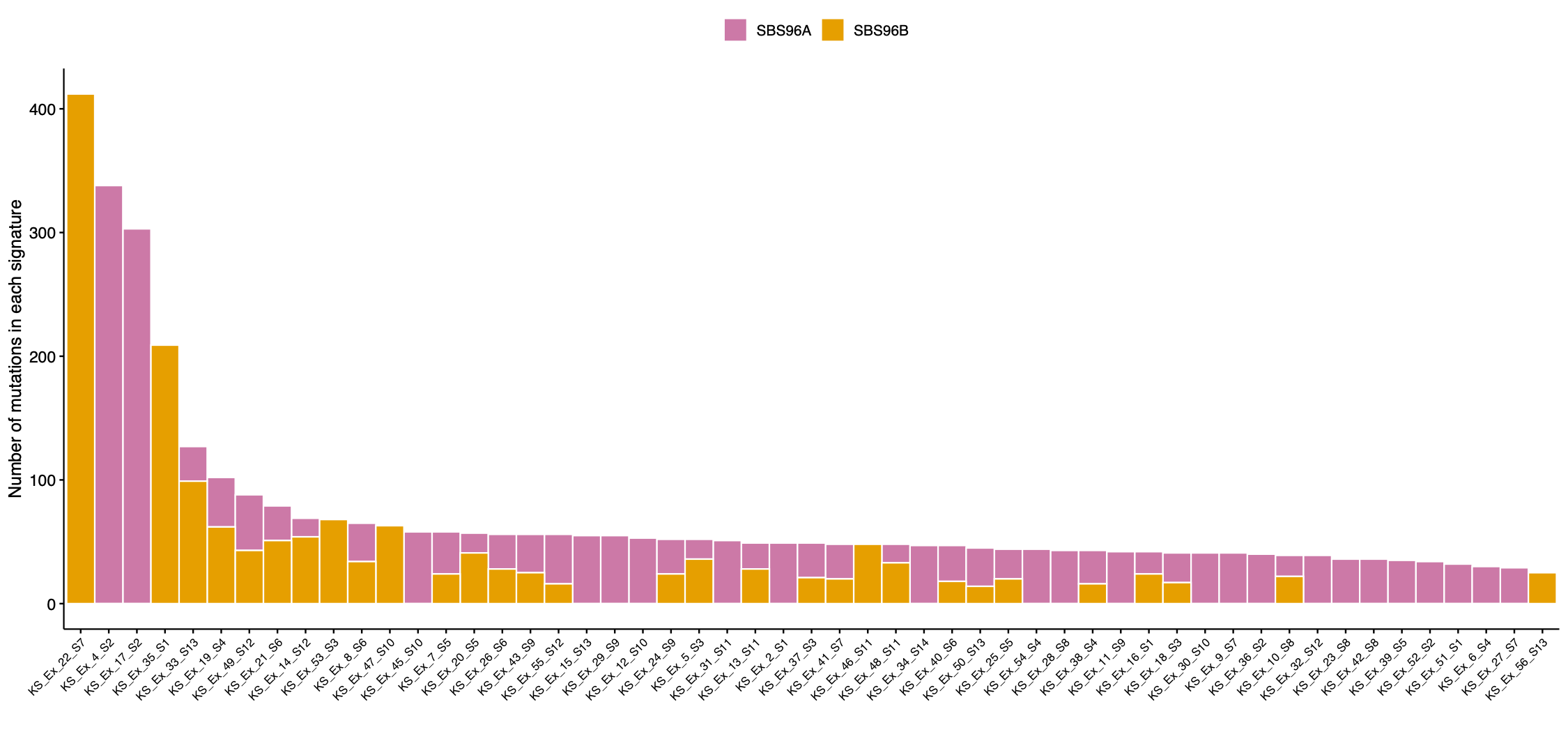
 NET cohort samples. The sample names are shown on the x-axis, and the number of pathogenetic or unknown variants in each signature is shown on the y-axis. The colours indicate which signature had the pathogenetic or unknown variants and which signatures were found in each sample.


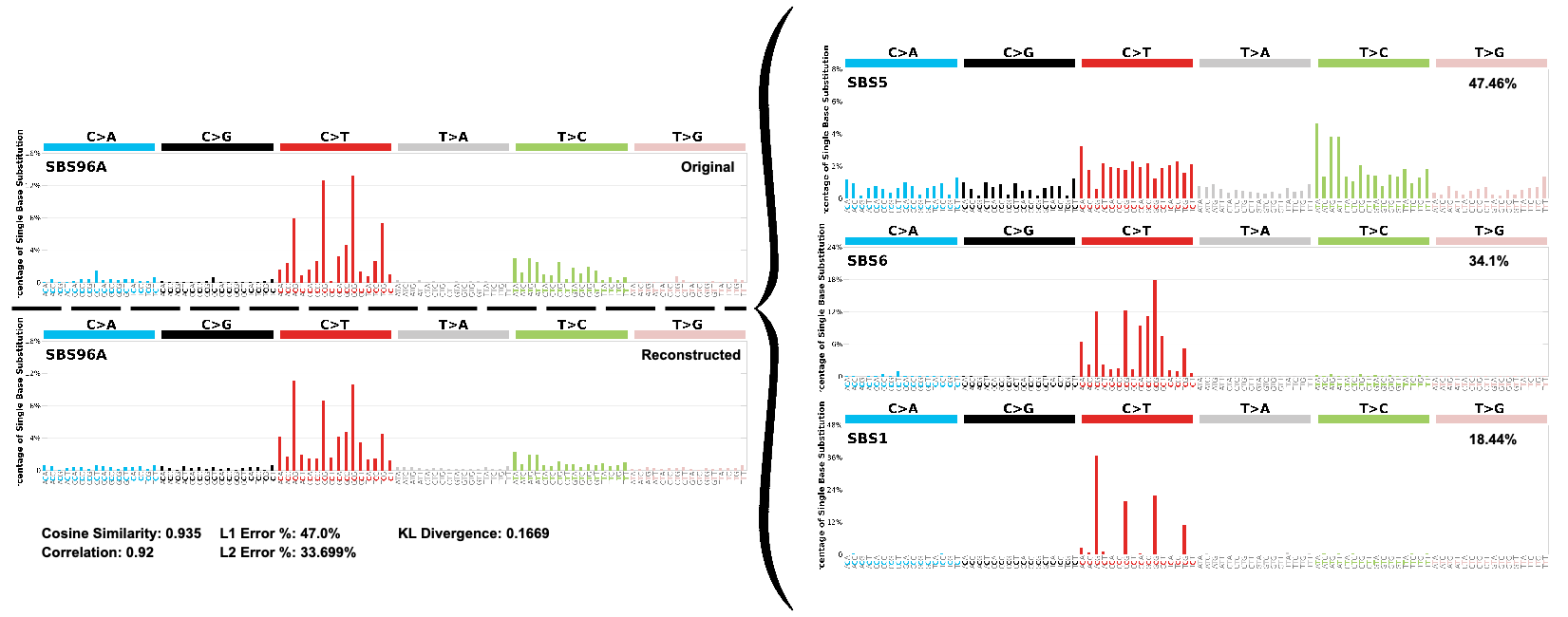

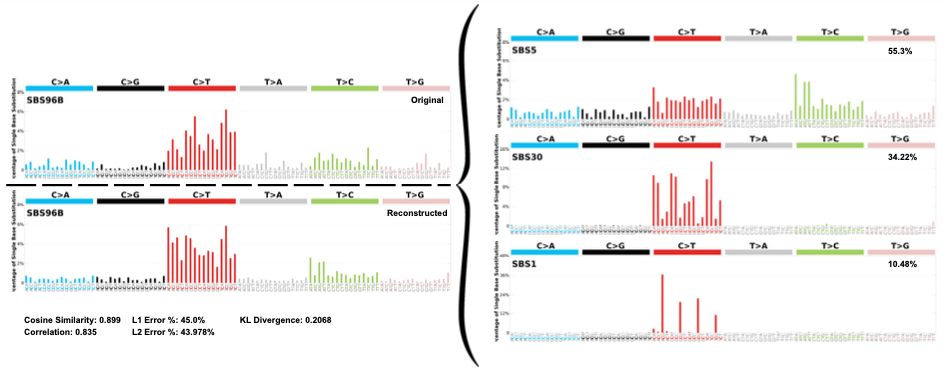


Supplementary Figure 6. SBS96 signature decomposition plots of the two de novo SBS signatures identified by SigProfiler from the breast NET cohort analysis. The two plots on the left side of the curly brace represent the original de novo signature plot (top) and the reconstruction of the de novo signature (bottom). On the right side of the curly brace are the COSMIC signatures of which the reconstructed de novo signatures are composed.


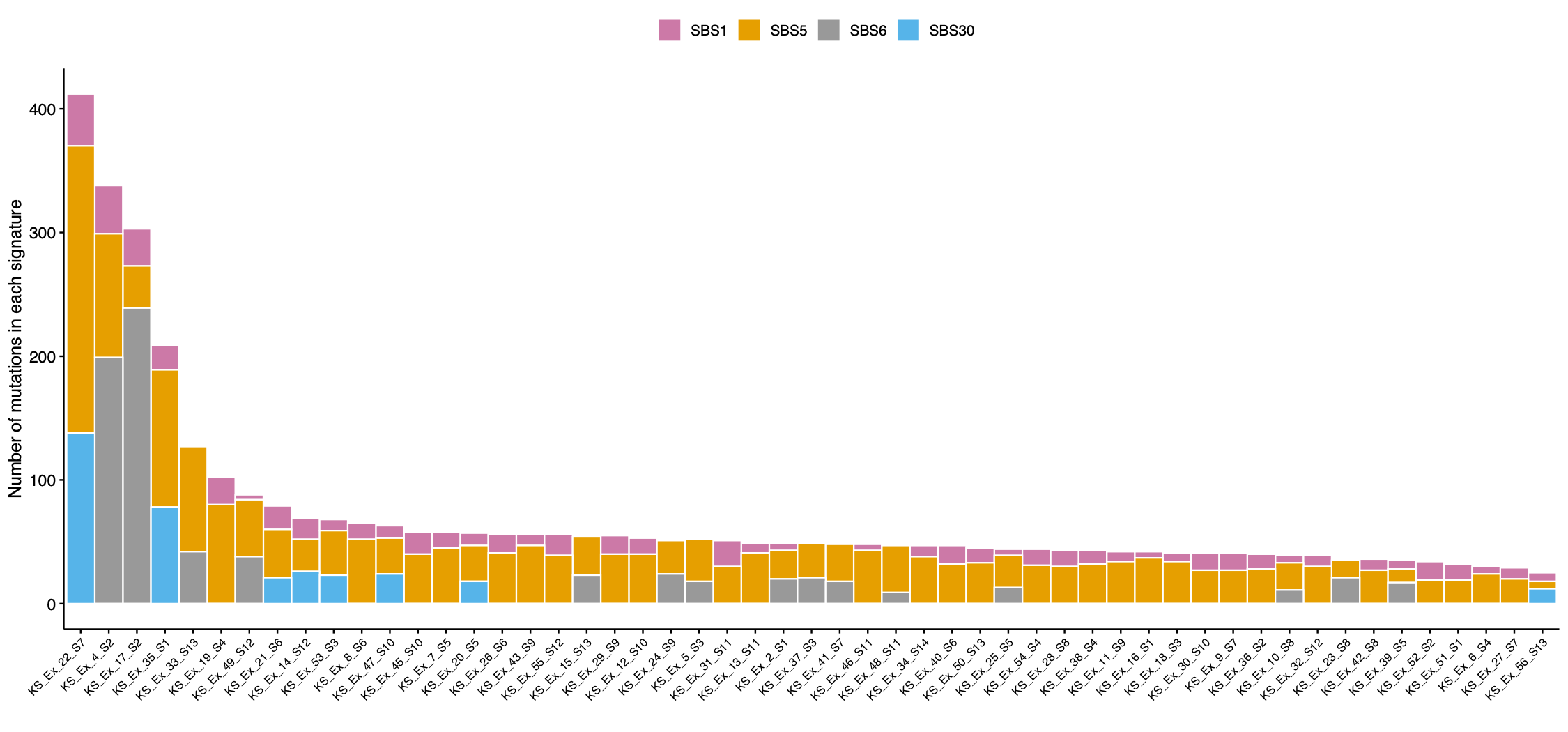


Supplementary Figure 7. Activity plot of the COSMIC SBS96 decomposed SigProfiler signatures in the breast NET cohort samples. The sample names are shown on the x-axis, and the number of pathogenetic or unknown variants in each signature is shown on the y-axis. The colours indicate which signature had the pathogenetic or unknown variants and which signatures were found in each sample.


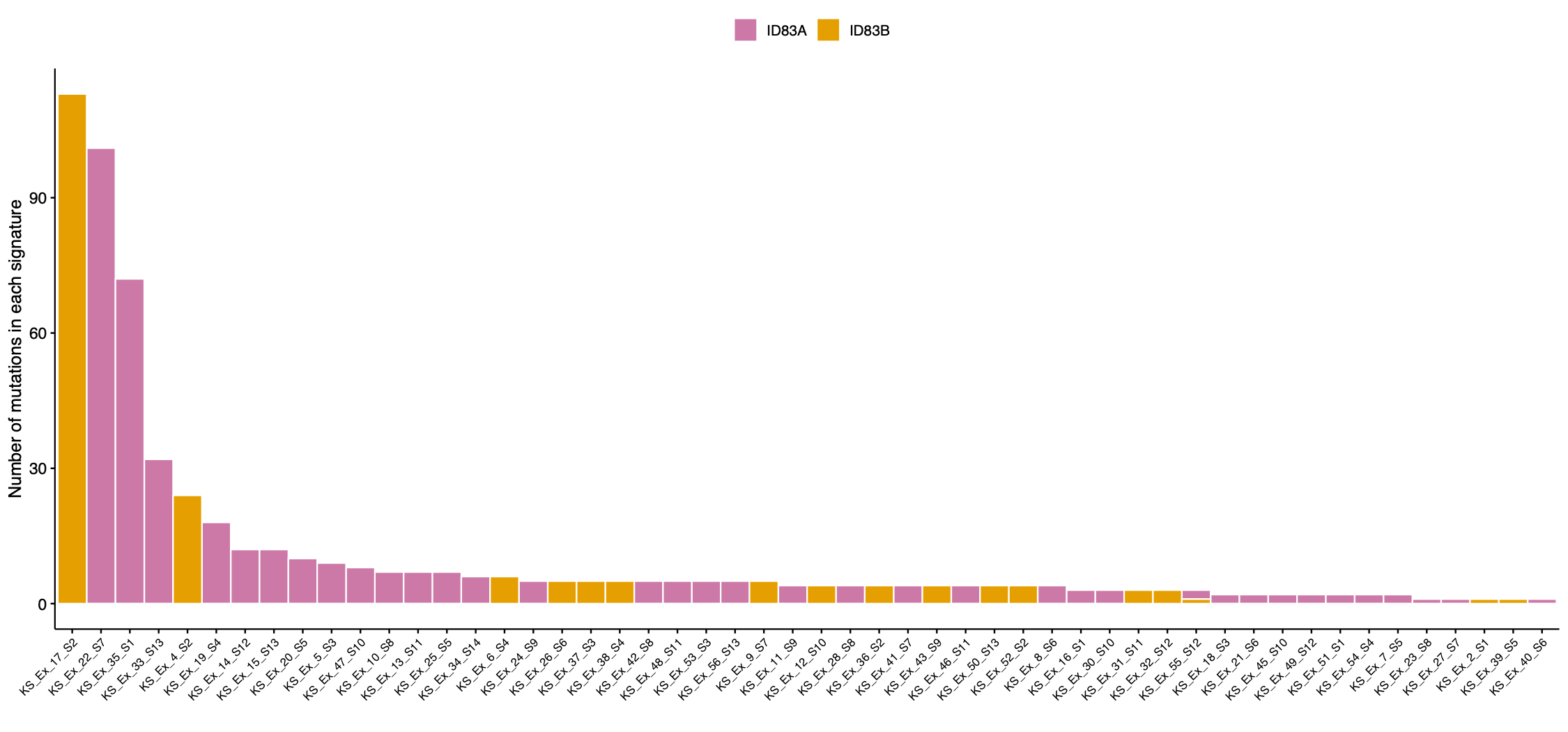

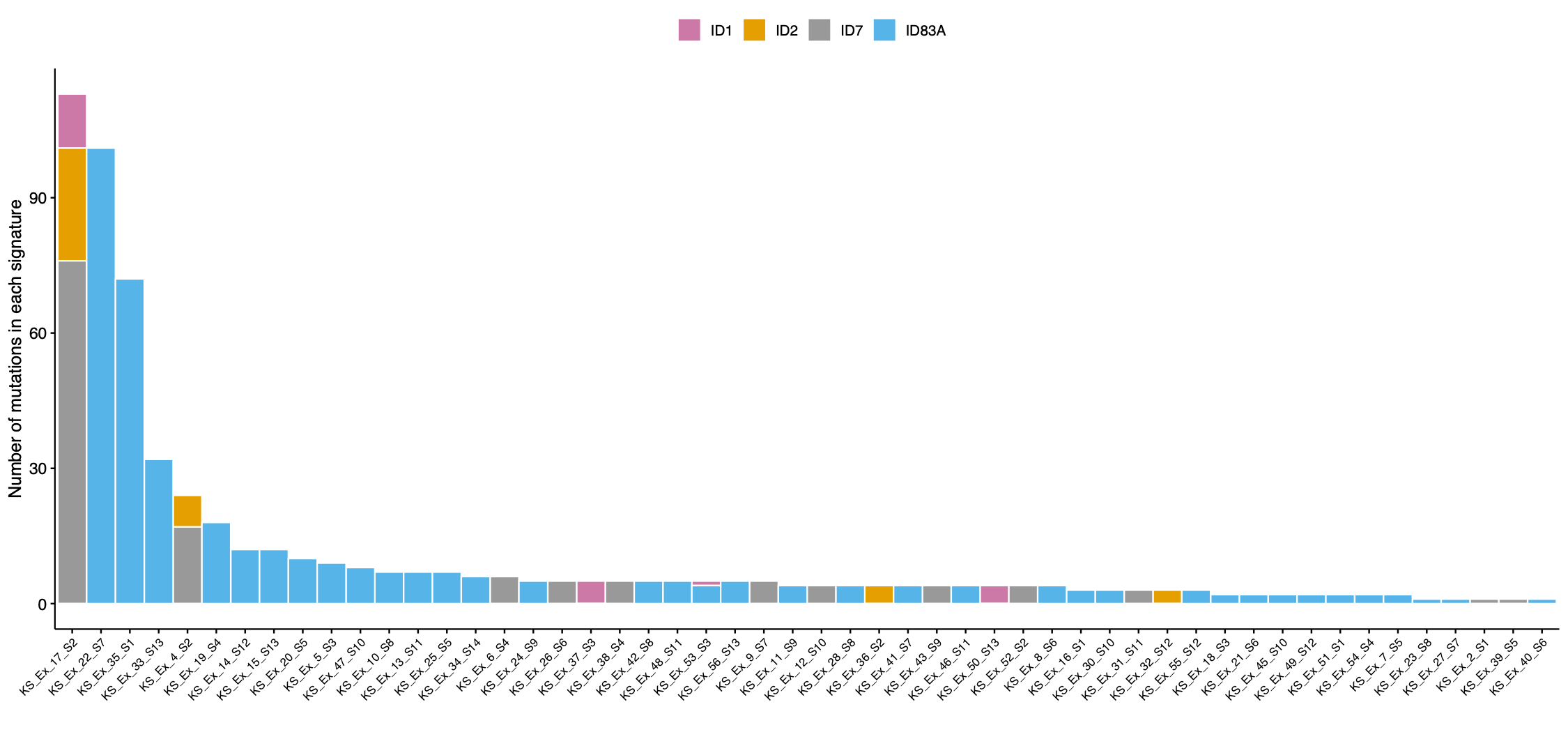


**A**

**B**

Supplementary Figure 8. Activity plots of the de novo ID83 (A) and decomposed COSMIC ID83 (B) signatures detected by SigProfiler in breast NET cohort samples. The sample names are shown on the x-axis, and the number of pathogenetic or unknown variants in each signature is shown on the y-axis. The colours indicate which signature had the pathogenetic or unknown variants and which signatures were found in each sample.


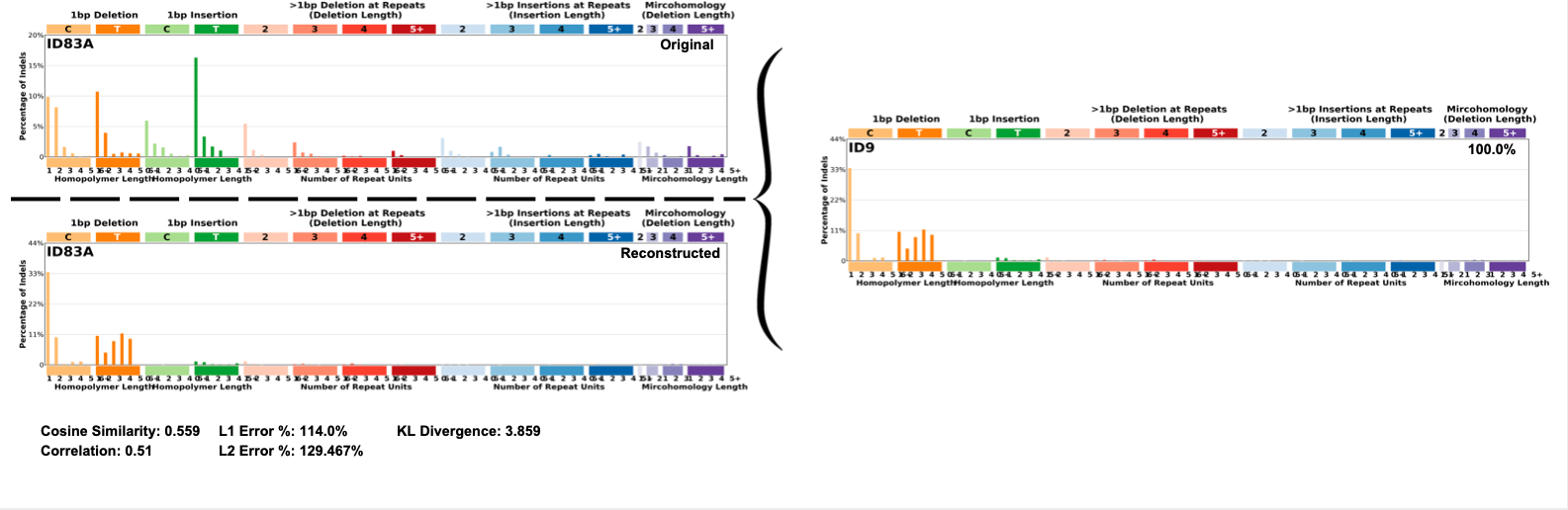

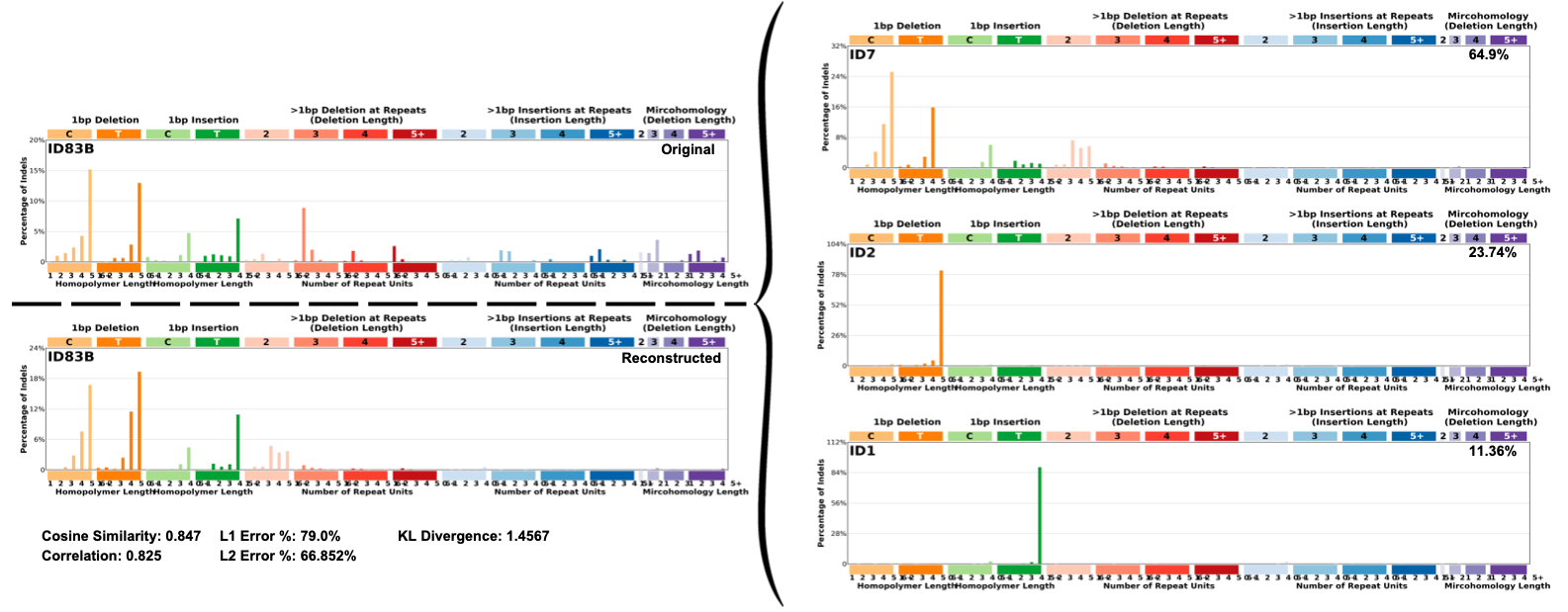


Supplementary Figure 9. ID83 signature decomposition plots of the two *de novo* ID signatures identified by SigProfiler from the breast NET cohort analysis. The two plots on the left side of the curly brace represent the original *de novo* signature plot (top) and the reconstruction of the *de novo* signature (bottom). On the right side of the curly brace are the COSMIC signatures of which the reconstructed *de novo* signatures are composed. Due to the low similarity between ID83A and its attempted decomposition (cosine similarity = 0.559), this signature was deemed novel.


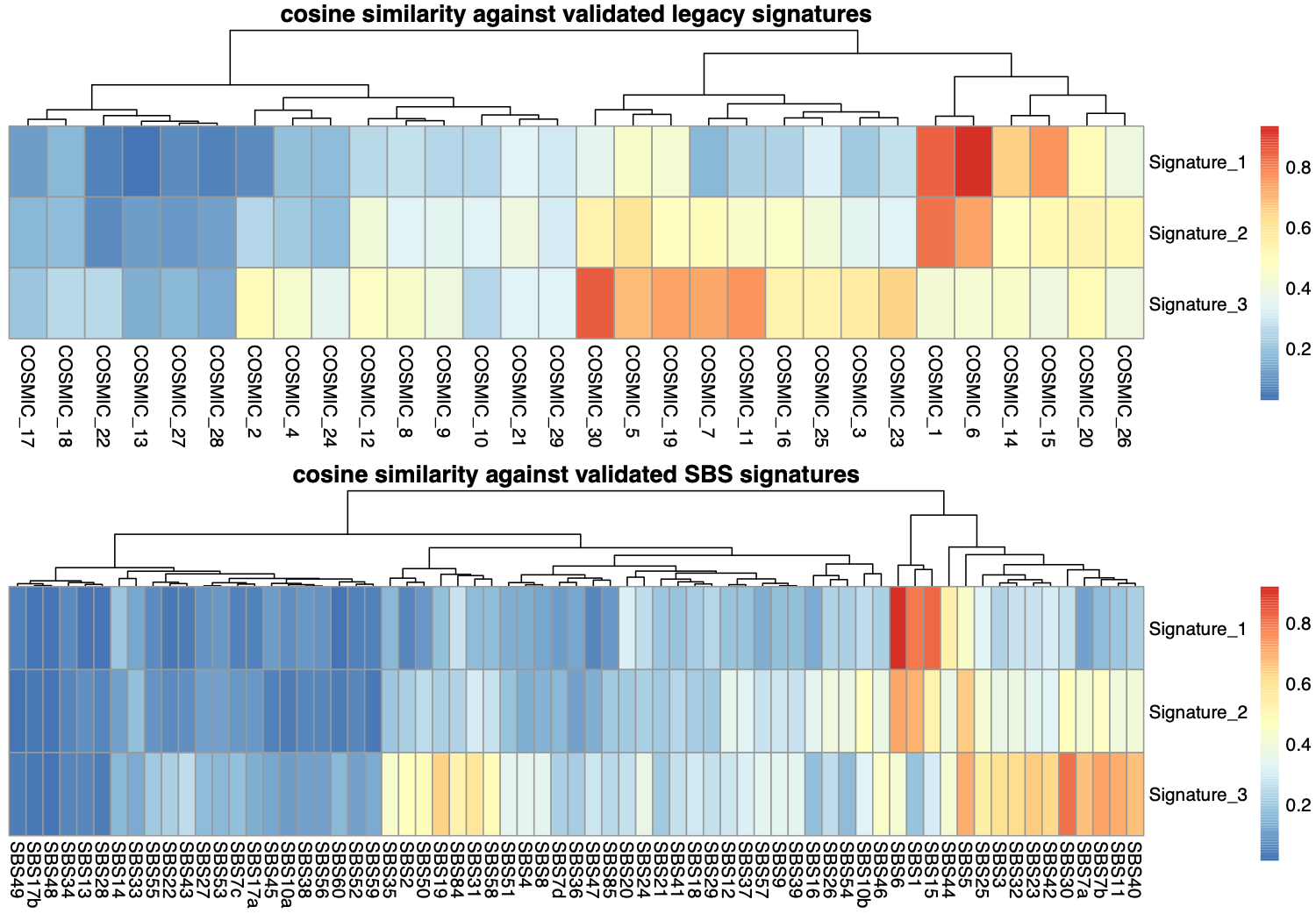


Supplementary Figure 10. Mutational signature analysis results from the analysis using Maftools represented as heat maps. The heat map at the top shows the cosine similarities of the three identified signatures to the 30 COSMIC legacy signatures, and the heat map at the bottom shows the cosine similarities of the three identified to the refined set of 65 SBS signatures.

Supplementary Figure 11. Mutational signatures identified by Maftools in breast NET samples and compared to COSMIC legacy signatures (A) and COSMIC SBS signatures (B). The y-axis indicates exposure of 96 trinucleot idemotifs to the overall signature. The best matches against the validated COSMIC signatures, cosine similarity values, and the proposed aetiology are shown above the plots.


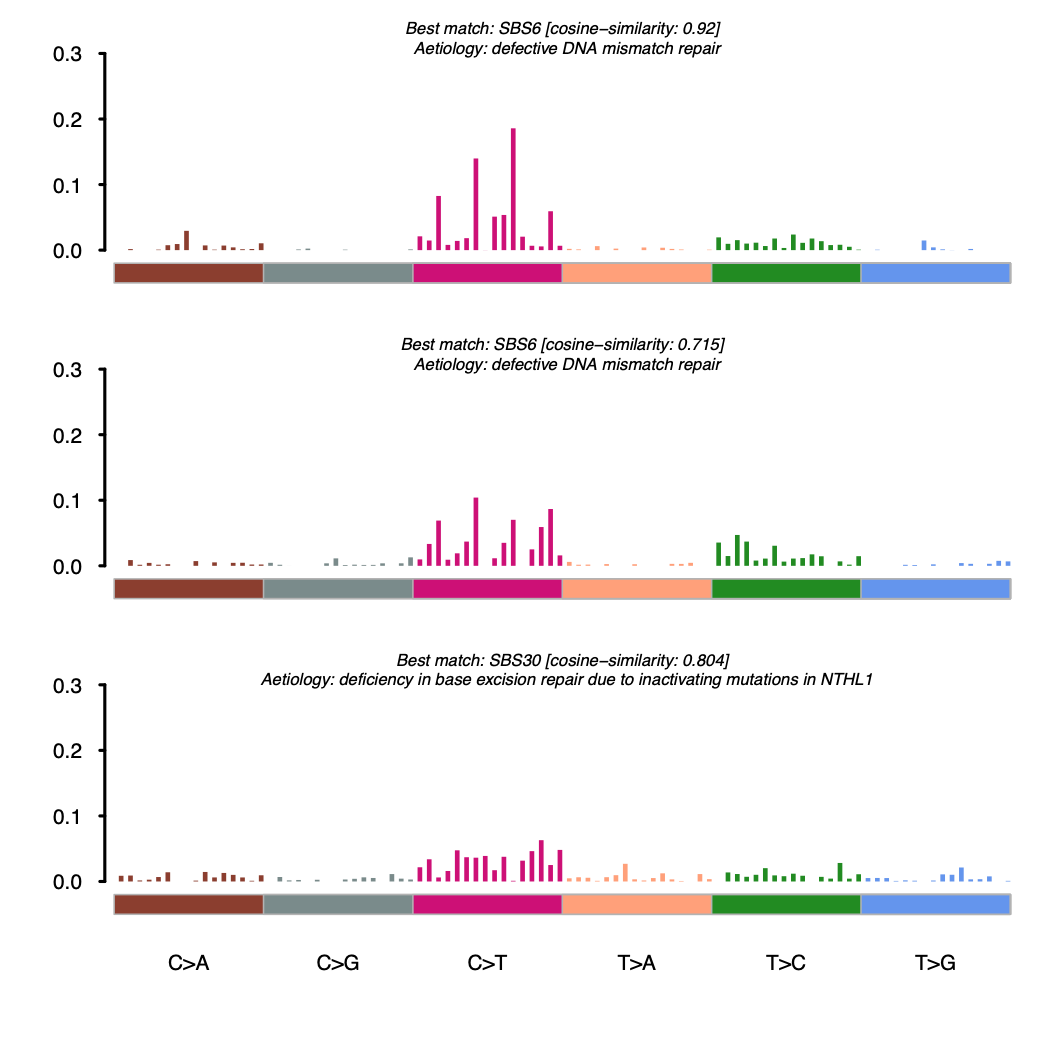

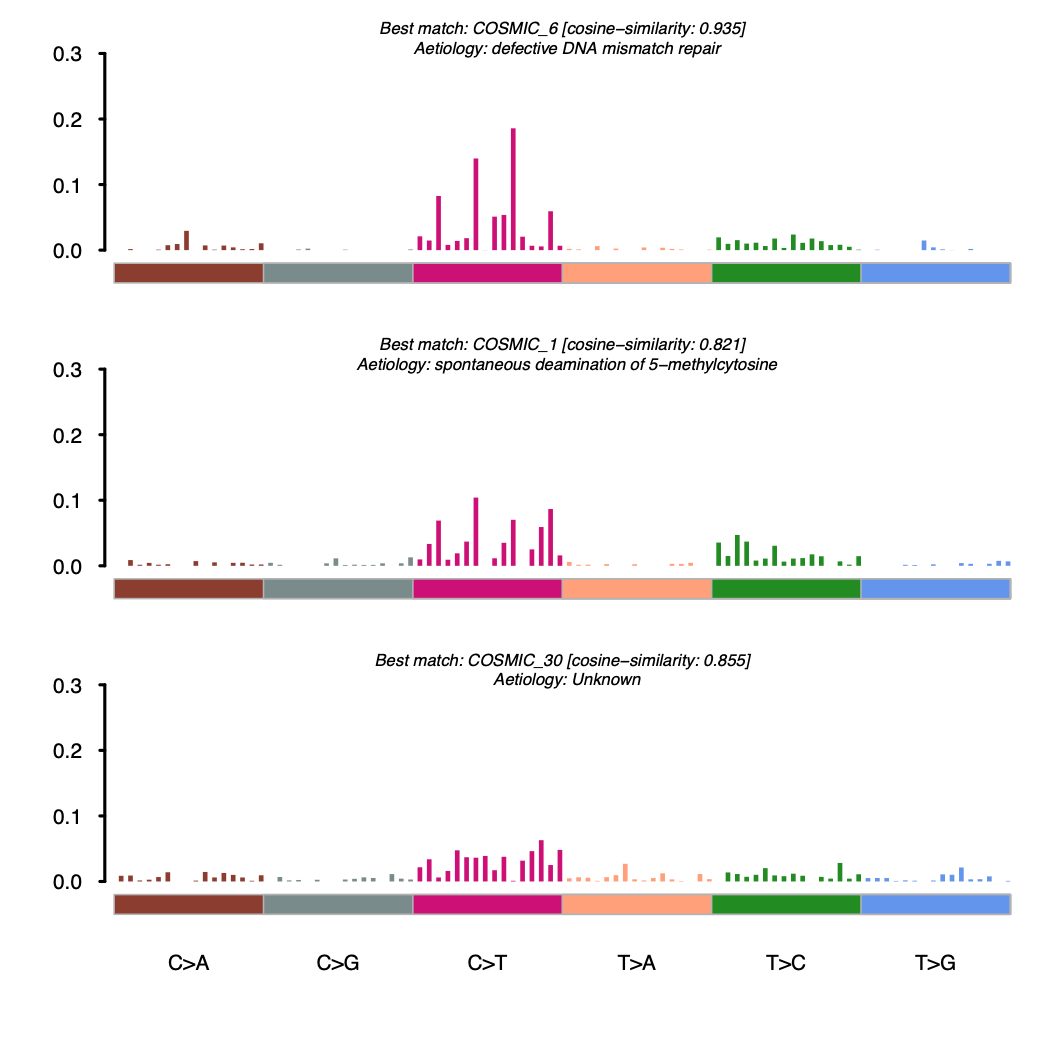


**A**

**B**


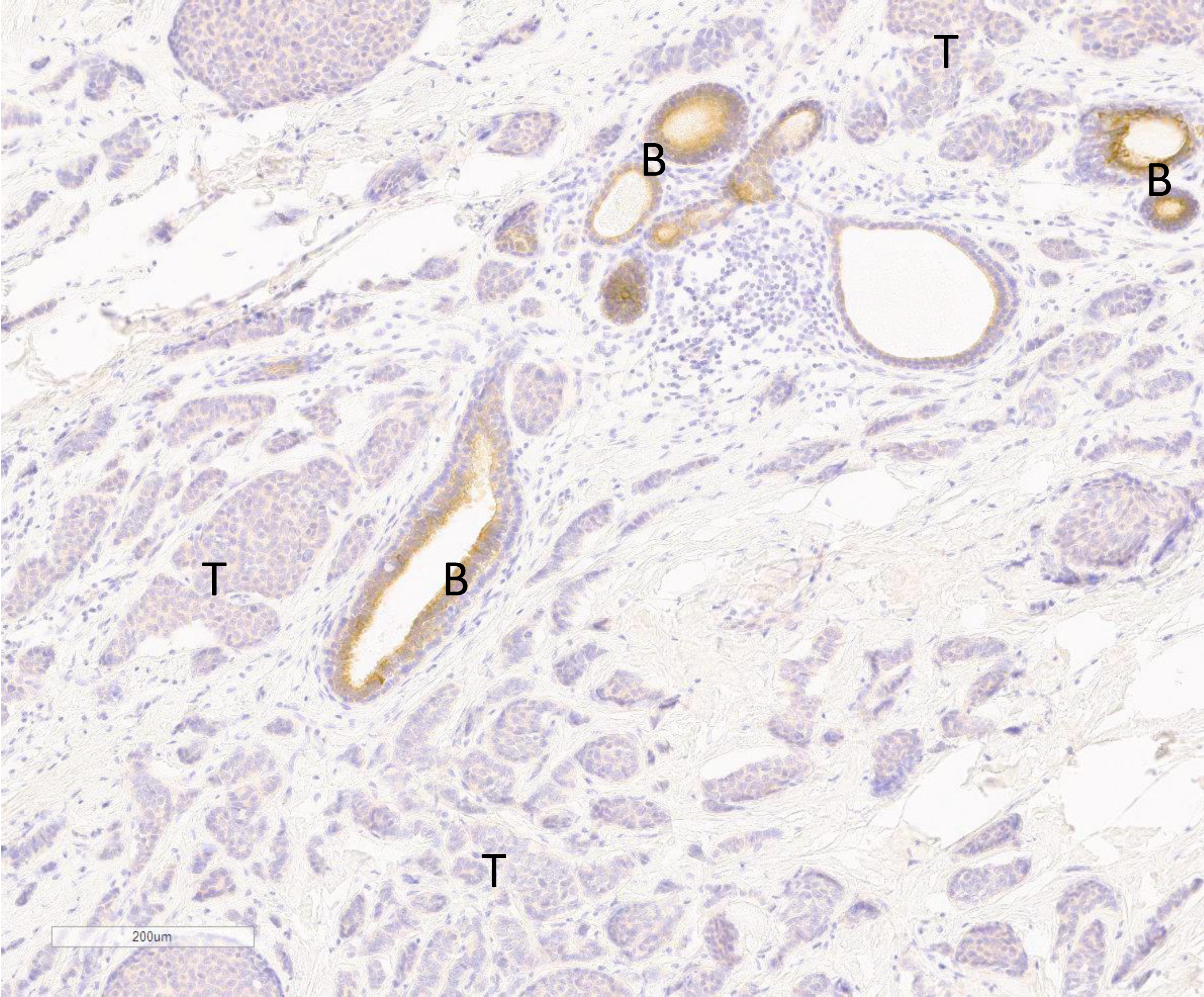


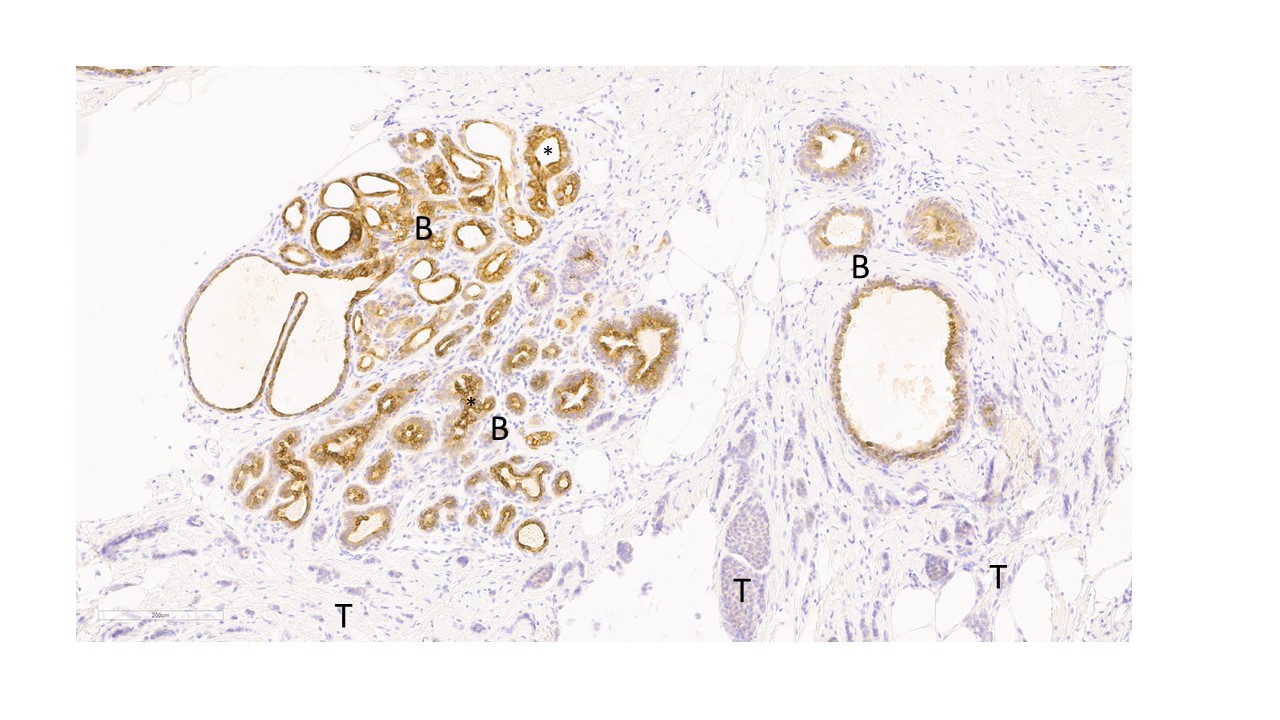


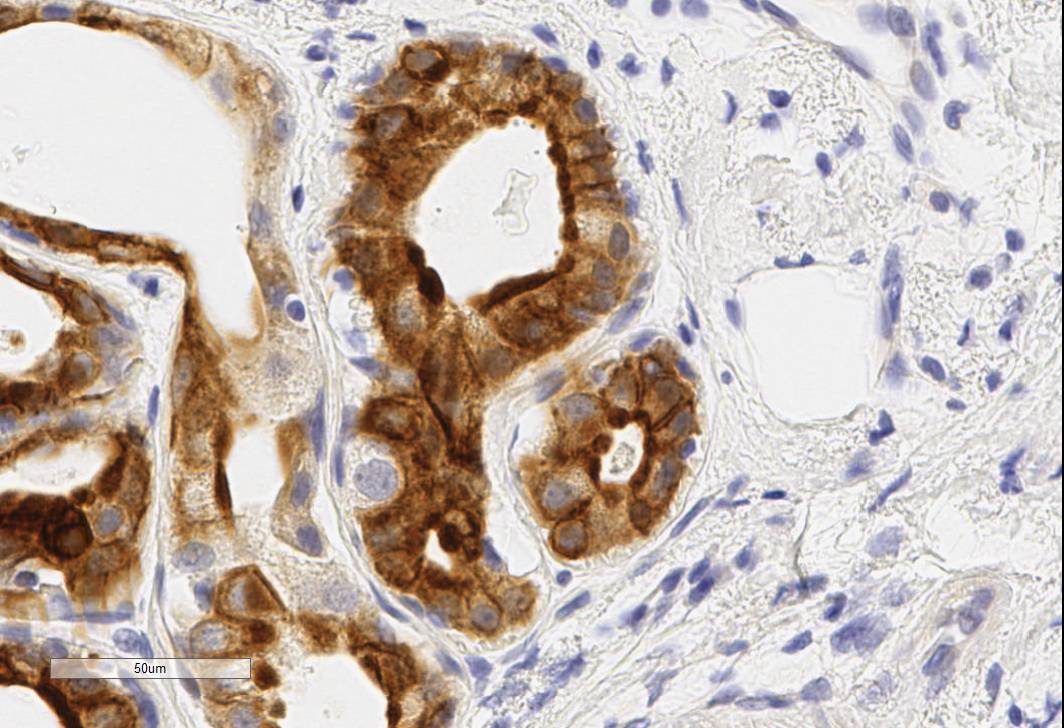


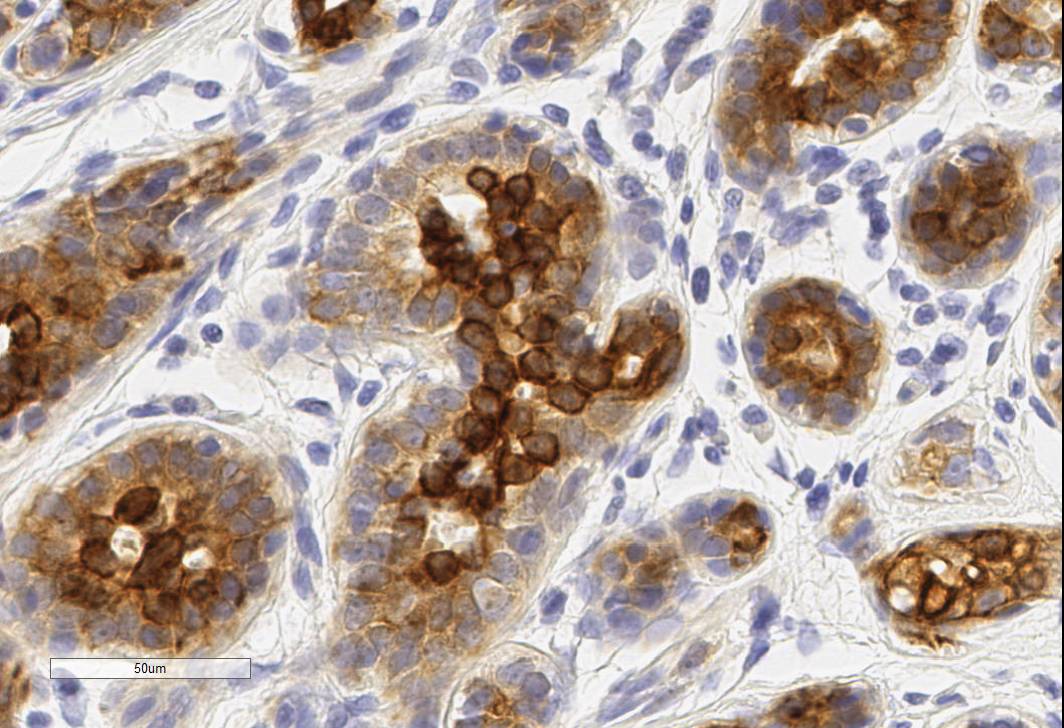


Supplementary Figure 12. Examples of ADCK2 immunohistochemical stainings in breast NET samples. The tumor tissue (T) is negative for ADCK2 antibody in both figures. A few of the non-affected acini in benign tissue (B) have strong membranous luminal staining and moderate cytoplasmic staining. Areas marked with asterics are shown in a higher magnification.


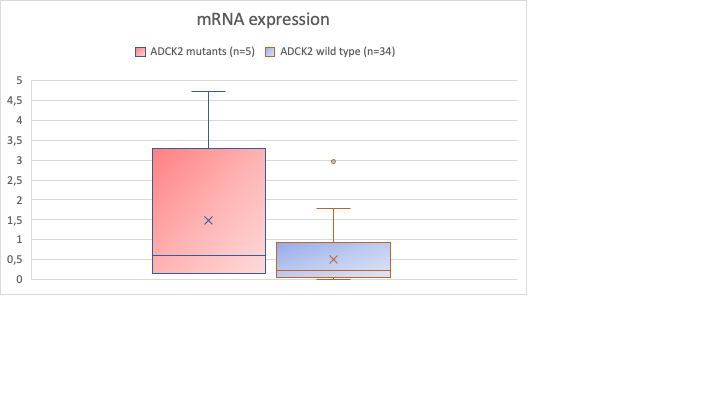


Supplementary Figure 13. ADCK2 mRNA expression was not statistically significantly different in the breast NET samples with the *ADCK2* pathogenetic or unknown variant.


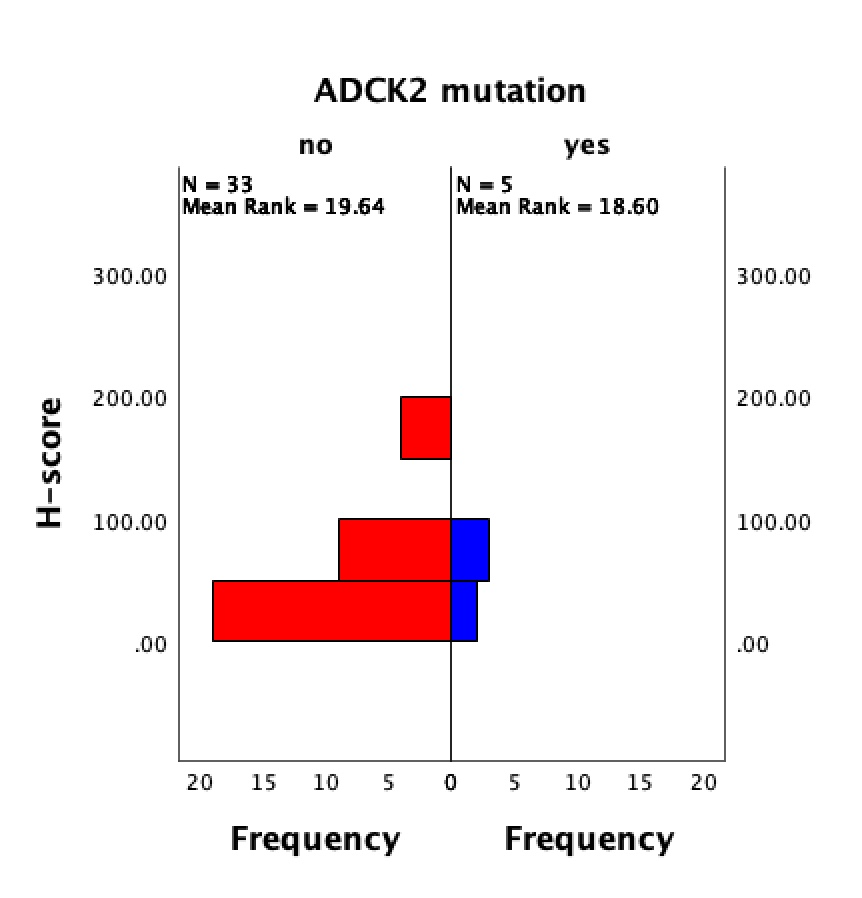


Supplementary Figure 14.

No association was observed between the mutational status of *ADCK2* and the immunohistochemically analysed ADCK2 protein expression (Mann-Whitney p=0.867). A histological sum score, the H score, was computed by multiplying the intensity and staining percentage scores, resulting in a scale of 0–300.

Pathogenetic or unknown variants associated with higher age at diagnosis: PCDH15 (p=8.6x10^-10^), SLC5A12 (p=8.6x10^-10^), ADGRL2 (p=2.1x10^-9^), FOXA1 (p=2.1x10^-9^), PLK4 (p=2.1x10^-9^), TTC37 (p=2.1x10^-9^), ULK2 (p=2.1x10^-9^), ITGA6 (p=2.0x10^-9^), GNB1 (p=0.00024), GOLGB1 (p=0.00024), TK1 (p=0.00024), RING1 (p=0.00082), MICAL2 (p=0.0038), DICER1 (p=0.016), H1-4 (p=0.016), EXOC4 (p=0.019), MAP2K7 (p=0.024), CCDC88C (p=0.040), PTK2B (p=0.040)

Pathogenetic or unknown variants associated with lower age at diagnosis: TACC2 (p=6.0x10^-10^), TRAPPC11 (p=8.6x10^-10^), WDR1 (p=8.6x10^-10^), ASH1L (p=2.5x10^-9^), DNAH17 (p=1.5x10^-8^), NRXN1 (p=1.0x10^-7^), CAMKK1 (p=6.6x10^-7^), SBNO1 (p=6.6x10-^7^), ADCY8 (p=3.1x10^-6^), MLH3 (p=4.1x10^-6^), NEMP1 (p=4.1x10^-6^), ANOS1 (p=4.1x10^-6^), VRK2 (p=0.00015), PRDM10 (p=0.00022), PPP2R1B (p=0.00025), CDK20 (p=0.00052), ACLY (p=0.00082), APOB (p=0.00082), BCL6 (p=0.00082), CDH23 (p=0.00082), EFTUD2 (p=0.00082), HUWE1 (p=0.00082), IFIH1 (p=0.00082), LAMB2 (p=0.00082), PSMD1 (p=0.00082), SF3A1 (p=0.00082), SIX2 (p=0.00082), STK31 (p=0.00082), NPR2 (p=0.0019), HDAC5 (p=0.00082), ARFGAP2 (p=0.0070), LARS1 (p=0.012), ADAMTSL2 (p=0.016), ADAMTS13 (p=0.019), AP1B1 (p=0.019), KDM3B (p=0.019), ROR1 (p=0.019), SORCS2 (p=0.019), ZNF592 (p=0.019), CLTC (p=0.025), EPHA4 (p=0.033), ITPR1 (p=0.033), PLEKHA7 (p=0.033)
